# Molecular mechanisms of phytoconstituents from selected Egyptian plants against non-small cell lung cancer using integrated in vitro network pharmacology and molecular docking approach

**DOI:** 10.1007/s00210-025-03834-4

**Published:** 2025-01-31

**Authors:** Lamiaa A. Shaala, Diaa T. A. Youssef, Mahmoud A. Ramadan, Azza A. Khalifa, Reham S. Ibrahim, Fred Valeriote, Ismail Celik, Hend M. Dawood

**Affiliations:** 1 Suez Canal University Hospital, Suez Canal University, Ismailia, 41522 Egypt; 2 Department of Natural Products, Faculty of Pharmacy, King Abdulaziz University, 21589 Jeddah, Saudi Arabia; 3 Department of Pharmacognosy, Faculty of Pharmacy, Suez Canal University, Ismailia, 41522 Egypt; 4 Department of Pharmacognosy, Faculty of Pharmacy, Assiut University, Assiut, Egypt; 5 Department of Pharmacognosy, Faculty of Pharmacy, Alexandria University, Alexandria, Egypt; 6 Henry Ford Health System, Department of Internal Medicine, Josephine Ford Cancer Center, Detroit, MI 48202 USA; 7 Department of Pharmaceutical Chemistry, Faculty of Pharmacy, Erciyes University, Kayseri, 38039 Turkey

**Keywords:** Amaryllidaceous alkaloids, Disk diffusion assay, Non-small cell lung cancer, Network pharmacology, Molecular docking, Gene ontology

## Abstract

Non-small cell lung cancer (NSCLC) is a widespread highly malignant type of lung cancer. Conventional chemotherapeutic drugs may be accompanied by both drug resistance and serious side effects in patients. Therefore, safer and more effective medications are urgently needed for the treatment of NSCLC. This study investigates the mode of action of 21 phytoconstituents previously isolated from the Amaryllidaceous plants *Crinum bulbispermum* (Burm.f.), *Pancratium maritimum* L., and *Hippeastrum vittatum* Herbert alongside the Asteraceous plant *Centaurea scoparia* Sieb. for therapy of NSCLC via in vitro cytotoxic, network pharmacology, and molecular docking analyses. Despite the in vitro and in vivo cytotoxic studies carried out on phytoconstituents from these plants in treating numerous cancer types, scarce information documenting their cytotoxic activity towards NSCLC cells is available. First, the compounds were tested for their in vitro cytotoxic activities and selectivity on human non-small cell lung cancer cells using disk diffusion assay. Compounds having significant potencies were promoted for network pharmacology analysis. Pharm mapper, Genecards, STRING, and KEGG databases were utilized for surfing target genes and pathways for these compounds, while for construction of compound-target-pathway (C-T-P) network, Cytoscape 3.7.1. freeware was used. Molecular docking and dynamics simulation were run for the top hit constituents against the most enriched molecular targets followed by in silico ADMET studies using Schrodinger^®^ suite and Gromacs. In vitro cytotoxicity testing demonstrated that crinamine was the most potent compound followed by lycorine, hemanthidine, and haemanthamine. The network pharmacology approach revealed the enrichment of acetyllycoramine, pluviine, 5-hydroxy-7-methoxy-2-methylchromone, and ismine. Whereas, androgen receptor (AR), epidermal growth factor receptor (EGFR), and estrogen-sensitive receptor alpha (ESR1) were the most enriched target genes. Pathway analysis revealed that central carbon metabolism, EGFR tyrosine kinase inhibitor endocrine resistance, and non-small cell lung cancer were the most enriched cancer-related pathways. Ismine possessed the most stable ligand-protein interactions when docked to the three proteins, with MD simulations further confirming its strong and consistent binding to AR, moderate stability with ESR-1, and lower stability with EGFR over the 100 ns trajectory. ADMET study conducted on the above compounds confirmed their excellent drug-likeness properties, oral bioavailability, and safety profiles highlighting the need for some structural modifications to pluviine to enhance its oral bioavailability. These integrated approaches showed that some constituents from the investigated plants interact synergistically against non-small cell lung cancer-related genes and pathways.

**Supplementary Information:**

The online version contains supplementary material available at 10.1007/s00210-025-03834-4.

## Introduction

Lung cancer is a malignant tumor that causes one of the highest morbidity and mortality rates among all cancer types worldwide. It is classified into small-cell lung cancer and non-small-cell lung cancer (NSCLC), the latter being more common and more malignant than small-cell lung cancer. Early diagnosis of NSCLC can be helpful in treatment, often by surgical removal of the tumor. However, most cases are diagnosed in late stages; thus, alternative therapies are recommended as chemotherapy, targeted therapy, or immunotherapy (Li et al. 2021). Conventional chemotherapeutic drugs may be accompanied by possible drug resistance and serious side effects in patients, that in turn worsen the patient’s condition. Therefore, safer and more effective therapies are urgently necessary for the treatment of NSCLC (Li et al. 2021).

The use of medicinal plants has gained momentum in developing countries and is considered a first-choice therapeutic approach in 80% of developing countries (Obaidullah et al. 2021). For centuries, many pharmacological effects of medicinal plants and their methods of administration have been documented; however, limited knowledge concerning the compounds responsible for their activity was available (Obaidullah et al. 2021) (Plant-food-derived bioactives in managing hypertension: from current findings to upcoming effective pharmacotherapies). In recent years, many compounds isolated from traditional medicine have proved effective as anti-tumor drugs (Chen et al. 2020); hence, the use of herbal medicine in conjunction with chemotherapeutic drugs can reduce adverse effects and alleviate patient suffering (Obaidullah et al. 2021) (New insights into the anticancer therapeutic potential of maytansine and its derivatives).

The Amaryllidaceae family is a group of monocotyledonous plants constituting more than 60 genera of which are *Crinum*, *Pancratium*, and *Hippeastrum* genera. Plants of the genus *Crinum* are perennial bulbous herbs that are widely distributed in temperate and subtropical regions (Kianfé et al. 2019). Traditionally, many *Crinum* extracts were reported for their analgesic and anti-inflammatory effects in addition to their wide use in rheumatism, earache, edema, swelling, and various inflammatory processes (Refaat et al. 2011; Fennell and Staden 2001). The genus *Pancratium* comprises about 15 species, distributed throughout the Mediterranean, African, and Asian regions. The extracts of the bulbs and flowers of *P. maritimum* were believed to have analgesic, antifungal, anticancer, purgative, hypotensive, emetic, and anti-inflammatory effects (Youssef et al. 2022).

Many members from both genera were phytochemically investigated due to their richness in active alkaloids and non-alkaloidal constituents (Refaat et al. 2009). The alkaloids of the Amaryllidaceae family are divided into six characteristic isoquinoline alkaloids, including lycorine type, galanthamine type, cherylline type, crinine type, and tazettine type alkaloids. Non-alkaloidal components include coumarins, sterols, aldehydes, acids, esters alcohols, esters, amines, and amides (Maroyi 2016). These constituents are well-known for their pharmacological effects, including diaphoretic, antiviral, hypotensive (Fennell and Staden 2001) antimicrobial, anti-cholinesterase, and cytotoxic activities (Kianfé 2020).

The genus *Centaurea*, family Asteraceae, contains several herbaceous species distributed mostly in the Middle East and Western Asia (Reda et al. 2021). Traditionally, many of the *Centaurea* species have been utilized in the treatment of cancer and microbial infections and as antidiabetic and antirheumatic. *C. scoparia* is an annual or biannual herb growing in Sinai, Egypt. It contains phenolic compounds including flavonoids and lignans as well as chlorinated and non-chlorinated guaianolides such as chlorojanerin, diain, janerin, and deacylcynaropicrin (Ahmed and Kamel 2014; Youssef and Frahm 1994; Youssef and Frahm 1998).

Despite the in vitro and in vivo cytotoxic studies carried out on isolates from these plants in treating numerous cancers including breast cancer, leukemia, adenocarcinoma, melanoma, and hepatic cancer, scarce information documenting their cytotoxic activity towards NSCLC cells is available (Lamoral-Theys et al. 2010; Koutová et al. 2021), hence our particular interest for discovering new potent and selective cytotoxic drugs from these isolates for our NSCLC treatment regimen with potential oral bioavailability and safety profiles.

Due to the difficulties in studying the in vivo mechanisms of tumorigenesis, alternative cell-based assays such as the disk diffusion soft agar colony formation assay were able to measure the ability of cells to proliferate in semi-solid matrices, hence became a cornerstone of our drug discovery research. It affords a rigorous tool to examine the efficacy of novel compounds or treatment conditions on cell proliferation and growth. Moreover, quantitative readings from the assay—presented as a zone of colony growth inhibition—can be utilized to evaluate differences in cellular tumorigenicity in case of comparison between control and treatment groups (Horibata et al. 2015).

There is no doubt that understanding the molecular mechanism by which plant extracts act on certain molecular targets is challenging owing to their complex nature, synergistic effects of their chemical constituents, and their multi-targeted mechanism of action (Taha et al. 2020). Recently, network pharmacological analysis has been employed for the prediction of the protein targets and the related disease pathways of plant active constituents through the construction of networks made of constituents, genes, pathways, and disease nodes. Network pharmacology applies the concept of “network target, multicomponent therapeutics,” which simulates the complex matrices of medicinal plants (Shawky 2019).

To verify the correlations between active compounds and potential target genes from network pharmacology analysis, molecular docking studies are often conducted. Molecular docking aims to identify potential molecular interactions at the binding site of proteins, especially after the increased number of new molecular targets owing to the completion of the human genome project, as well as the use of protein purification techniques such as X-ray crystallography and nuclear magnetic resonance spectroscopy techniques (Majumder et al. 2016). It is deemed to be a useful tool for the identification of the most common ligand-protein binding mode(s) (Hasan et al. 2022). The technique generates a so-called docking score that is widely used for hit identification during computer-aided drug design (Lee et al. 2020). In drug discovery, the successfulness of a certain drug is not disclosed to its pharmacological activity, but it should also bear acceptable safety, potency, and pharmacokinetic profile (James et al. 2023). Many tools are employed for predicting the ADMET properties of compounds, among which is QikProp which uses several physical and pharmacological descriptors to predict pharmaceutically related properties of biomolecules and afford ranges to compare their scores with those of 95% of known drugs (James et al. 2023). It is a crucial step to prevent the failure of a potential lead molecule in clinical studies (Saini et al. 2022).

The present study was designed to elucidate the in vitro cytotoxic activity of some compounds isolated from *C. bulbispermum*, *P. maritimum*, *H. vittatum*, and *C. scoparia* to NSCLC cells using a disk diffusion assay. The most promising phytoconstituents will be forwarded to network pharmacology interpretation aiming to unveil the multi-component synergistic mechanism of the plant constituents in treating NSCLC and pinpoint the related protein targets and pathways. The top-scored compounds will be docked into the active sites of the most enriched target proteins to investigate the potential ligand-receptor interactions and assess their binding affinities.

## Materials and methods

### Isolation of compounds

**Fig. 1:**
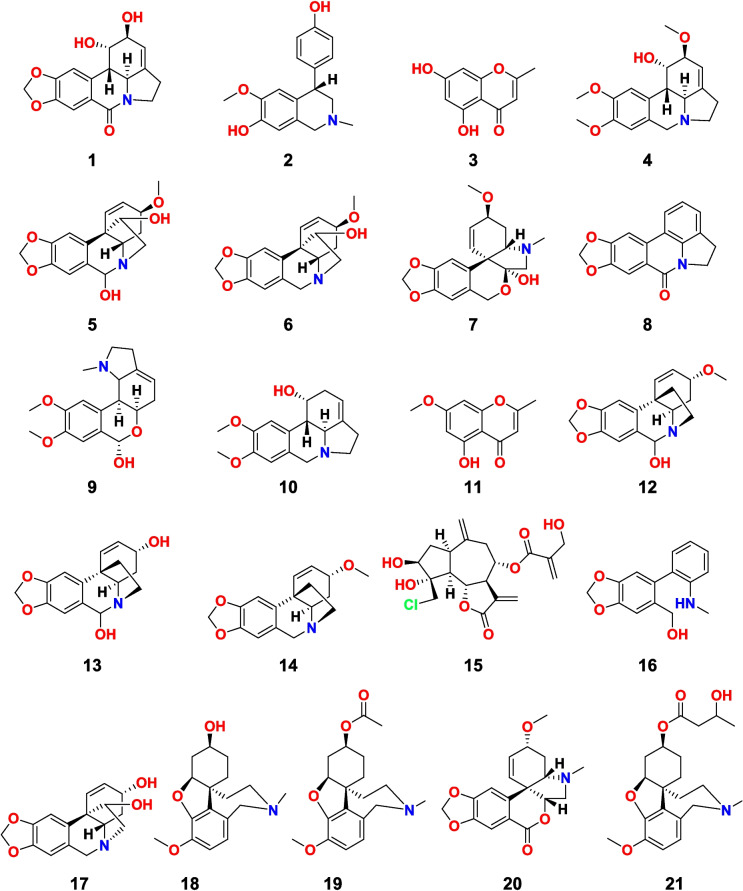
Chemical structures of compounds **1**–**21**

Twenty-one compounds were previously isolated from Egyptian plants belonging to different genera of Amaryllidaceae and Asteraceae families using previously adopted procedures and were characterized using different spectral analyses. Comparing the compounds’ spectra to those reported in the literature confirmed their identities. Lycorine (**1**), cherylline (**2**), galanthine (**4**), hemanthidine (**5**), haemanthamine (**6**), tazettine (**7**), hippadine (**8**), lycorenine (**9**), pluviine (**10**), 6-hydroxybuphanisine (**12**), 6-hydoxycrinine (**13**) and buphanisine (**14**), crinamine (**17**), and macronine (**20**) were isolated previously by our team from the bulbs of *C. bulbispermum* (Ramadan 1986). 5,7-dihydroxy-2-methylchromone (**3**) (Ali et al. 1990), 5-hydroxy,7-methoxy-2-methylchromone (**11**) (Ali et al. 1990), acetyllycoramine (**19**) (Youssef 1999), and pancritamine (**21**) (Youssef 1999) were isolated by us from the bulbs of *P. maritimum*. Further, lycoramine (**18**) (Youssef and Frahm 1998) was reported by us from the flowers of *P. maritimum*, while ismine (**16**) was purified from *H. vittatum (*Youssef 2001). Finally, chlorojanerin (**15**) was obtained by us from the flowering aerial parts of *C. scoparia* (Youssef and Frahm 1994; Youssef and Frahm 1998). The chemical structures of these compounds are illustrated in Fig. 1.

### In vitro disk diffusion assay

The disk diffusion assay defines the differential cell killing between the solid tumor, human lung H125, and both a human leukemia cell, CCRF-CEM, and a murine and human normal cell (hematopoietic progenitor cell, CFU-GM). Differential cytotoxicity is the endpoint. The use of human cell lines was approved by the Ethical Committee of Alexandria University, Alexandria, Egypt (Approval No: 062023720342A).

Human CCRF-CEM is the reference leukemia tumors of the assay and is used as a basis to define samples that are differentially cytotoxic to solid tumor cells, thereby defining solid tumor selective samples in which the active compound’s structure and activity can be expected to be quite different from many standard anticancer agents which were initially defined by the leukemia screens. CCRF-CEM is a human T-cell lymphocytic leukemia originally cultured from a 3-year-old patient in 1965 (Foley 1965) obtained by us from ATCC and has been adapted to growth in our agar assay. It has a long history of use by investigators (and is in the NCI 60-cell panel) as a sensitive leukemia model.

The second reference cell type chosen is a normal cell, CFU-GM, the granulocyte/macrophage committed progenitor stem cell from the marrow, represents a target cell for the most common chemotherapeutic toxicity in vivo, namely myelosuppression. The use of a normal cell population to compare with the cancer cell lines used in this in vitro assay platform is a critical feature and can give early insights into selectivity and predicted therapeutic indices. We initially ran murine CFU-GM from Swiss mice. For the human solid tumor samples selective against murine CFU-GM, we rerun with human CFU-GM (Lonza Walkerville, Inc., MD) for a definitive outcome. Cells are used for about 30 passages and then re-started from frozen stock to minimize the effect of any long-term culture genetic drift (Torsvik et al. 2014). The cells are examined every 5 years and tested for a genetic match using the ATCC and DSMA reported profiles. For plating of all cell types other than the normal CFU-GM, the 60-mm plates are first prepared with a hard agar bottom layer (0.6% agar in RPMI-1640 plus 20% BCS). Further, for plating of H125, a soft agar top layer (0.3% agar with the serum and media as above) plus the titrated tumor cells are poured onto the solidified hard bottom agar layer in the plates and allowed to solidify. The human cancer cell lines are maintained in cell culture. They are removed from their cultures by trypsin, 0.05% (Hyclone, Utah); 30,000 cells in 3 mL produce over 2000 colonies per plate (60-mm plates).

For CFU-GM, the femoral marrow of Swiss mice is flushed with MEM-alpha, 2 mL per femur. The cells are passed through an 18-gauge needle twice, and the monodispersed suspension is counted. A total of 1.5 × 10^6^ cells are plated in 3 mL of 0.3% agar with the addition of 10% L-cell conditioned media, which provides a colony-stimulating factor, in MEM-alpha plus 10% BCS. Human CFU-GM cells are received from overnight delivery and washed twice with PBS before being titered and added to the agar mixture. The same cell number, culture conditions, and conditioning factors are used as with the murine marrow plus additional conditioning factors: 1% L-glutamine, 1% Pen-Strep, 1% β-mercaptoethanol, 1.6 ng/mL granulocyte-macrophage colony-stimulating factor (Peprotech, NJ), and 1.6 ng/mL recombinant M-CSF (Stem Cell Technologies, Vancouver, BC).

Lung-H125 is an adenosquamous carcinoma from an untreated 61-year-old male that was developed by the NCI-Navy Medical Oncology Branch (Sherwin et al. 1981; Phelps et al. 1996).

The step-by-step laboratory methods for our in vitro assay have been described (Corbett et al. 1992; Valeriote et al. 2012; Valeriote et al. 2002). In brief, A volume of 15 µL of each sample is dropped onto a 6.5-mm filter disk (Millipore). The disk is dried overnight and then placed close to the edge of the petri dish. The plates are incubated for 7 days and examined by an inverted stereo microscope (× 10) for measurement of the zone of inhibition measured from the edge of the filter disk to the beginning of normal-sized colony formation. The diameter of the filter disk, 6.5 mm, is arbitrarily taken as 200 units. A zone of less than 200 units is taken as the extract is of insufficient activity to be of further interest. A difference in zones between solid tumor cells and either normal or leukemia cells of 250 units defines solid tumor selective compounds (Valeriote et al. 2012; Valeriote et al. 2002). If the test material is excessively toxic at the first dosage, we then retest a range of dilutions of the agents (at 1:4 decrements) against the same tumors. At some dilution, quantifiable cytotoxicity is invariably obtained. The results are expressed as, for example, _H125_*Δ*_CFU_ = 350, which indicates that there is a 350-unit zone differential between human lung H125 and normal CFU-GM; such a differential (> 250 units) is of a sufficient magnitude to proceed with fractionation if the sample were an extract or to proceed with further cellular and pharmacology studies if the sample were a pure compound. The principle of the disc diffusion assay method is illustrated in (Fig. S1) in the supplementary materials.

The synergistic effect between the most active compounds was also investigated using the same protocol of zone diffusion assay on the combination.

### Network pharmacology analysis

#### Preparation of compounds for network analysis

The 2D structures of the isolated active compounds were drawn using ChemDraw Professional 17.1 (PerkinElmer) and converted to the SMILES format using the Online SMILES Translator (https://www2.chemie.uni-erlangen.de/services/translate/).

#### Target genes of constituents related to NSCLC

SwissTargetPrediction database (https://labworm.com/tool/swisstargetprediction) and PharmMapper Server (http://www.lilab-ecust.cn/pharmmapper/) were utilized to identify potential target gene candidates for the compounds with the “*Homo sapiens*” species setting. The structures of compounds were imported into both of them to match the targets. GeneCards database (https://www.genecards.org/) provided comprehensive information on all annotated and predicted human genes related to NSCLC. Venny2.1.0 database (https://bioinfogp.cnb.csic.es/tools/venny/) was used to screen the candidate targets related to both active compounds and NSCLC by finding the common genes in the intersecting area of Venn diagram. Protein–protein interaction network (PPI network) was constructed using STRING database (https://string-db.org/). UniProt (http://www.uniprot.org/) (Chandran and Patwardhan 2017; Shi et al. 2019) was utilized for retrieving gene information including name, gene ID, and accession number.

#### Networks construction

Constituent-target genes pathway (C-T-P) network was constructed using Cytoscape 3.7.1 (https://cytoscape.org/download.html). In the graphical representation, each constituent, target gene and pathway was described by node, and the interactions were encoded by edges. The network parameters were calculated using the network analyzer plug-in. The P-P interaction diagram was constructed using the STRING online database.

### Gene ontology enrichment analysis

Database for Annotation, Visualization, and Integrated Discovery (DAVID) ver. 6.8 (https://david.ncifcrf.gov/) and the Kyoto Encyclopedia of Genes and Genomes (KEGG) pathways database (http://www.genome.jp/kegg/pathway.html) were utilized to gain information about gene ontology (GO) and for the addition and identification of the canonical pathways, biological processes, cellular components, and molecular functions that were strongly related to the target genes.

### Molecular docking studies

The 3D structures of top-scored compounds were imported as SDF into the LigPrep module of Maestro 10.2 molecular modeling (Schrödinger, LLC, New York) software package to obtain low-energy structures of compounds. Ionization at pH 7 was performed to produce all possible state discoveries of potential natural dihydroorotate dehydrogenase inhibitors and their synergism with Brequinar via integrated molecular docking. High-resolution crystal structures of the top 3 enriched targets revealed from network pharmacology analysis named AR, EGFR, and ESR-1 were retrieved from Protein Data Bank (PDB ID: 2PIW, 1M17, and 6CBZ, respectively). Protein preparation was accomplished using the protein preparation module of Maestro where they were preprocessed by assigning bond orders and hydrogens in addition to removing all the water molecules beyond 5 Å from the active site. Assignment of H-bonds was performed via PROPKA at pH 7, then energy minimization using OPLS 3 force field was performed till the relative mean standard deviation (RMSD) of the minimized structure compared to the crystal structure was above 0.30 Å. Biologically guided isolation of natural lead antithyroid drug from *Medicago sativa L*. sprout receptor grid generation module using boxes enclosing the centroids of co-crystallized ligands were set as the grids. Molecular docking analysis was achieved using the Glide docking program of the Maestro molecular modeling package implementing extra-precision (XP-Glide) mode and anticoagulant activity screening of an in-house database of natural compounds for discovering novel selective factor Xa inhibitors, a combined in silico and in vitro approach. The binding modes of the compounds with targets were visualized using the Maestro interface. Furthermore, the root mean square deviation (RMSD) for each crystalline structure was sought to further validate the docking protocol. This was accomplished by utilizing the pose selection method to re-dock the cocrystallized ligand into its designated binding site in each respective crystalline structure. Thereafter, the docked pose was compared to the crystal structure’s pose, and the RMSD was calculated (Synergistic effect of potential alpha-amylase inhibitors from Egyptian propolis with acarbose).

### Molecular dynamics simulation

Molecular dynamics (MD) simulations were performed to evaluate the dynamic stability and interaction patterns of ismine with AR (PDB ID, 2PIW), ESR-1 (PDB ID, 6CBZ), and EGFR (PDB ID, 1M17). The initial input files for the simulations were prepared using the CHARMM-GUI (Jo et al. 2008) web interface, with the CHARMM36m (Brooks et al. 2009) force field applied for parameterization. Each protein–ligand system was solvated in a TIP3P water box, ensuring a buffer of at least 10 Å around the complex. A total of 0.15 M KCl was added to neutralize the system and mimic physiological ionic conditions. Energy minimization was performed to remove steric clashes, followed by equilibration in multiple stages under an NVT ensemble with restraints applied to the heavy atoms. The systems were further equilibrated for 0.25 ns under an NPT ensemble to ensure proper density and pressure. The production MD simulations were carried out for 100 ns using Gromacs 2023.3 (Abraham et al. 2015) under periodic boundary conditions, with a time step of 2 fs. The Particle-Mesh Ewald (PME) method was used for long-range electrostatics, and a cutoff of 1.2 nm was applied for short-range non-bonded interactions. The temperature was maintained at 303.15 K using a velocity-rescale thermostat, while pressure was regulated at 1 bar using a Parrinello–Rahman barostat. All bonds involving hydrogen were constrained using the LINCS algorithm. Key analyses were performed on the MD trajectories, including root mean square deviation (RMSD) to assess stability, a radius of gyration (Rg) to evaluate compactness, and binding energy calculations using MMPBSA to quantify interaction strengths. The results provide a detailed insight into the dynamic behavior and stability of the protein–ligand complexes.

Binding free energy calculations molecular mechanics Poisson–Boltzmann surface area (MMPBSA) were performed using the g_mmpbsa tool (Kumari et al. 2014) on the final 200 frames extracted from the molecular dynamics simulations’ 80–100 ns interval. The binding energy components, including van der Waals, electrostatic, polar solvation, and SASA energy, were calculated to provide insights into the contributions to the total binding free energy.

### In silico ADMET study

ADMET properties of the top four hit molecules were determined by the QikProp module located in Schrödinger software. The compounds were first prepared and incorporated into the QikProp tool. Many ADMET descriptors were calculated including Lipinski’s rule of five, Predicted octanol/water partition coefficient (QPlogPo/w), predicted Caco-2 cell permeability (QPPCaco), Predicted binding to human serum albumin (QPlogKhsa), predicted aqueous solubility (QPlogS), number of hydrogen bonds donated (donorHB) and accepted (AccptHB), percentage human oral absorption, number of metabolic reactions (#metab), Jorgensen’s rule of three, and half maximal inhibitory concentration (IC_50_) value for human Ether-à-go-go-related gene potassium channel (QPlogHERG). This approach permits researchers to focus on certain metabolites that deserve further investigation.

## Results and discussion

### Zone assay results of isolated compounds

The disk diffusion soft agar colony formation assay could be used as a powerful tool in evaluating the efficacy of different drugs in mitigating the proliferation of cancer cells. This assay determines a zone of inhibition for a sample diffusing from a disk placed at a petri dish containing tumor cells embedded in a soft agar matrix. A decrease in colony formation with respect to an increase in drug concentration indicates effective cytotoxicity of the drug in vitro. It has an advantage over many assays as the semi-solid matrix permits a favorable environment for the anchorage growth of cancer cells which cannot be exhibited by normal cells as well as it mimics the in vivo 3D cellular environment (Horibata, 2015). In this study, 21 compounds isolated from *C. bulbispermum*, *P. maritimem*, *H. vittatum*, and *C. scoparia* were tested for their tumor growth inhibitory activities to NSCLC cells (H-125).

The zone assay was designed to look for selectivity between the human solid tumor, H125, and either the leukemia (CEM) or the normal cell (CFU-GM). It can also provide a measure of potency. Table 1 shows the initial concentration of the stock solutions of the compounds (column 2), the amount used from these stock solutions in the assay (column 3), and the zone of inhibition against H125, CEM, and CFU-GM (columns 4, 6, 7, respectively). A cutoff of 200 units/µg was used in this assay. Potency is defined as the ratio of the H125 zone and the amount of compound added to the filter disk (columns 6 and 7, respectively). Table 1 displays the potency of **1**–**21** in descending order with three distinct groups in this table. As shown in Table 1, group A comprises nine compounds (**17**, **1**, **5**, **6**, **16**, **10**, **4**, **11** and **15**) that were considered as active. Crinamine (**17**) was the most potent one among this group followed by lycorine (**1**), hemanthidine (**5**), and haemanthamine (**6**), and Group B comprises a set of four compounds (**19**, **12**, **20**, and **21**) that had H125 zones of between 100 and 200 zone units. In our studies, we usually call these as moderately active, since a cutoff of above 200 zone units was set for activity. Finally, group C with eight compounds (**2**, **3**, **7**, **8**, **9**, **13**, **14**, and **18**) is considered inactive because there are no zones of inhibition observed. Only one compound, pluviine (**10**), demonstrated any solid tumor selectivity, ^H125^*Δ*_CFU_ = 275 units.

**Table 1:** Disk diffusion assay results of compounds 1–21

Compound no	Initial conc	Dilution	Disc amt	H125 zone	Ratio	CEM	CFU-GM
	(mg/mL)		(µg)	(arbitrary unit)	(units/µg)	zone (arbitrary unit)	zone (arbitrary unit)
Group A							
17	4	1/64	0.94	500	532	350	< 700
6	8.5	1/16	7.9	> 1000	127	> 1000	900
1	10	1/64	2.3	600	261	650	500
5	5.8	1/16	5.4	750	139	650	700
16	5.5	1/4	21	350	17	500	< 600
10	4.2	1	63	725	11.5	650	450
4	5.5	1	83	500	6	400	400
11	5.5	1	83	450	5.4	250	450
15	10	1	150	700	4.7	850	800
16 + 17	5.5 + 4	1/4 + 1/64	21 + 0.94	> 1000	17 + 532	900	900
Group B							
19	5	1	75	200	2.7	250	ND
12	5.8	1	87	200	2.3	ND	ND
20	5	1	75	150	2	350	ND
21	5	1	75	100	1.3	ND	ND
Group C							
2	5	1	75	0			
3	9.5	1	143	0			
7	7.5	1	113	0			
8	5.2	1	78	0			
9	7	1	105	0			
13	6.8	1	102	0			
14	5	1	75	0			
18	5.5	1	83	0			

^*^Results are expressed as mean of three determinations

The synergistic effect is such an important criterion especially when considering candidates for cancer treatment (Bayat Mokhtari 2017). Therefore, an in vitro assay was also carried out to investigate the synergistic effect of crinamine (**17**) (being the most in vitro active compound among the tested candidates) and ismine (**16**) (the top hit as revealed from subsequent in silico studies). Surprisingly, this combination exhibited a synergistic effect on H-125 cells (Table 1). Where the H125 zones exceeded 1000 zone units for the combination, exceeding their individual additive zone units, 500 and 350 zone units for crinamine and ismine, respectively. Therefore, this approach promotes this combination for potential treatment of NSCLC.

### Network pharmacology analysis

#### Target fishing and construction of networks

To examine the profound pharmacological mechanisms of the most active isolates in treating NSCLC, interactions with the related targets and identification of involved pathways were investigated using “Network Pharmacology” analysis (Shi et al. 2019).

**Fig. 2:**
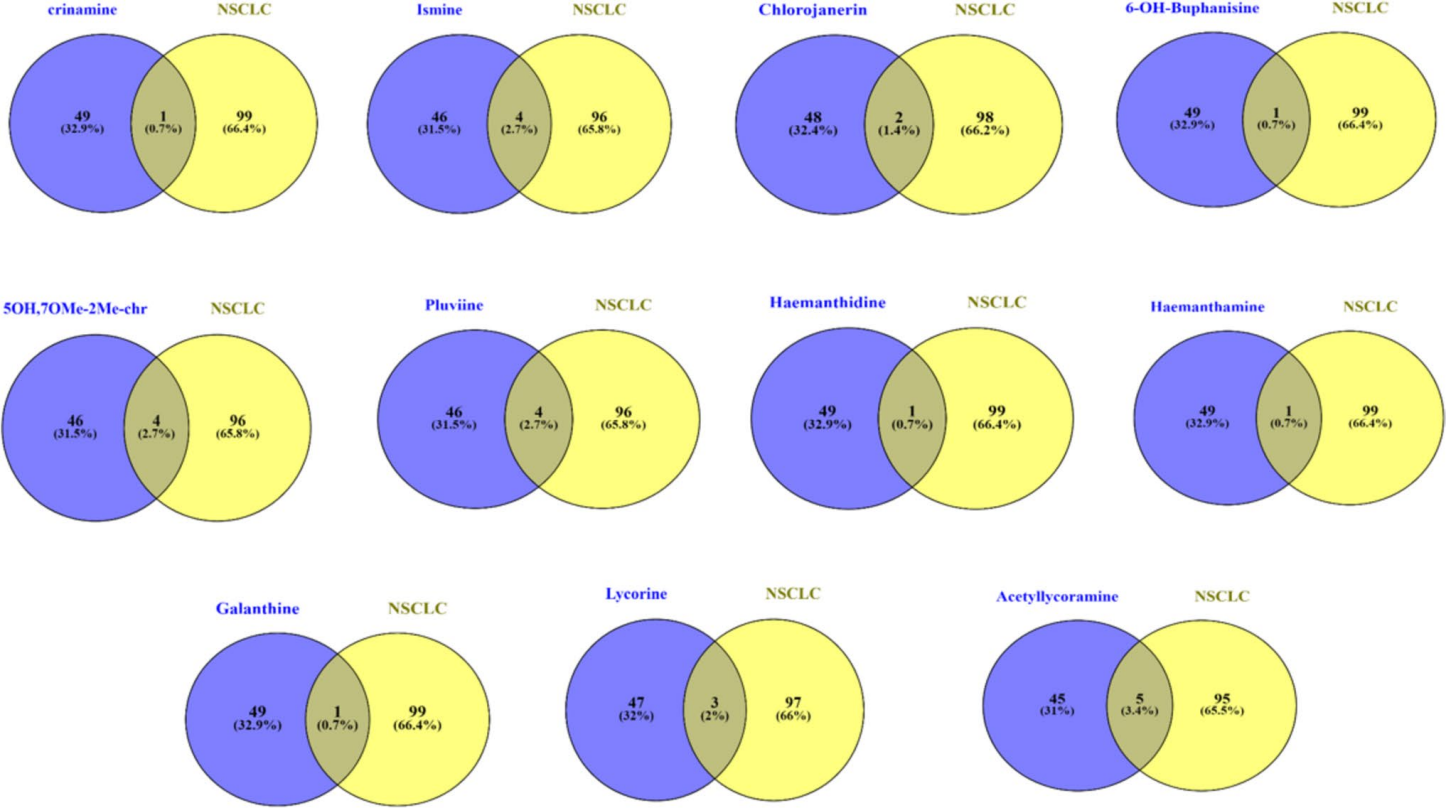
Venn diagrams illustrating the number of NSCLC-related genes shared by each of the top-scored compounds

The top 50 potential targets having the highest interaction combined scores for each of the active compounds were analyzed with the top 200 NSCLC-related targets using Venny 2.1.0 aiming to identify the possible common genes (Fig. 2, Table 2). Common genes varied from one compound to another, where acetyllycoramine (**19**) shared the highest number of overlapping genes with NSCLC (5 genes), followed by ismine (**16**), pluviine (**10**), and 5-hydroxy-7-methoxy-2-methylchromone (**11**) (4 genes), while crinamine (**17**) showed interactions with only one NSCLC-related gene.

**Table 2:** Information on potential NSCLC-related protein targets shared with the top scored isolated compounds

Target gene short name	Target gene full name	UNIPROT accession no	No. of interacting compounds	Name of interacting compounds
AR	Androgen receptor	P10275	6	Hemanthidine (**5**), haemanthamine (**6**), pluviine (**10**), 6-hydroxybuphanisine (**12**), ismine (**16**), crinamine (**17**)
EGFR	Epidermal growth factor receptor	P00533	4	Galanthine (**4**), pluviine (**10**), ismine (**16**), acetyllycoramine (**19**)
ESR1	Estrogen-sensitive receptor alpha	P03372	3	Lycorine (**1**), 5-hydroxy-7-methoxy-2-methylchromone (**11**), chlorojanerin (**15**)
ERBB2	Receptor tryrosine-protein kinase	P04626	2	Pluviine (**10**), ismine (**16**)
MAP2K1	Dual specificity mitogen-activated protein kinase 1	Q02750	2	Pluviine (**10**), ismine (**16**)
MET	Hepatocyte growth factor receptor	P08581	2	5-Hydroxy-7-methoxy-2-methylchromone (**11**), acetyllycoramine (**19**)
SRC	Proto-oncogene tyrosine-protein kinase	P12931	2	Lycorine (**1**), chlorojanerin (**15**)
ALK	ALK tyrosine kinase receptor	Q9UM73	1	5-Hydroxy-7-methoxy-2-methylchromone (**11**)
KIT	Mast/stem cell growth factor receptor Kit	P10721	1	5-Hydroxy-7-methoxy-2-methylchromone (**11**)
MTOR	Serine/threonine-protein kinase	P42345	1	Acetyllycoramine (**19**)
PIK3CA	Phosphatidylinositol 4,5-bisphosphate 3-kinase catalytic subunit alpha isoform	P42336	1	Acetyllycoramine (**19**)
PPARG	Peroxisome proliferator-activated receptor gamma	P37231	1	Lycorine (**1**)
TERT	Telomerase reverse transcriptase	O14746	1	Acetyllycoramine (**19**)

It did not escape our notice that the results obtained from network pharmacology analyses deviated slightly from those of the in vitro cytotoxic activity assay. This was probably due to the difference in pharmacokinetic profiles of the different compounds including hydrophobicity, solubility, molecular size, and weight that might significantly affect the penetration power of these compounds through the cancer cells to exert their cytotoxic activity. This prompted us to conduct further in silico pharmacokinetic studies to better understand their properties and provide suggestions for pharmaceutical and structural modifications to achieve enhancement in the compound’s permeability.

**Fig. 3:**
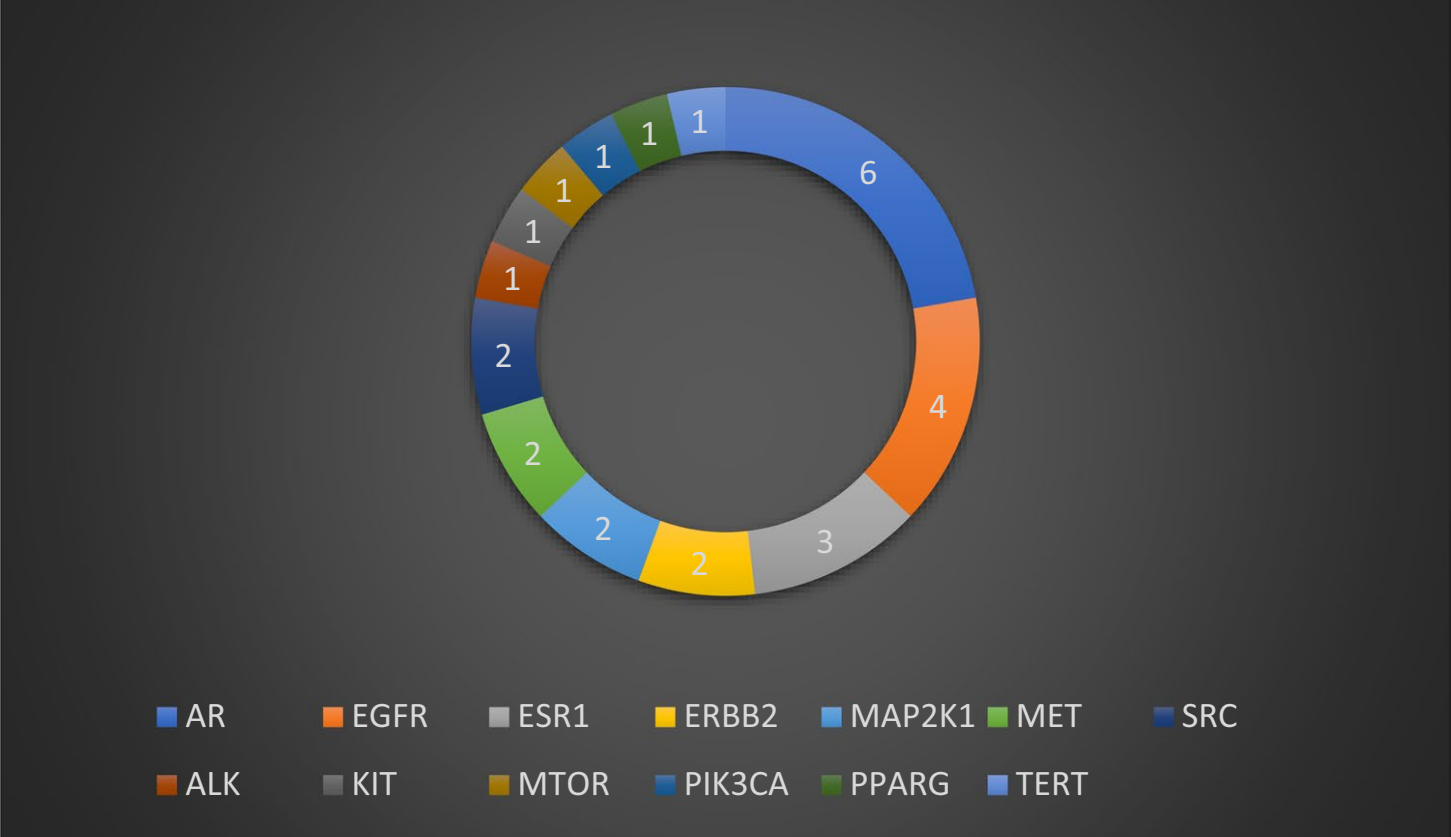
A donut chart showing the number of constituents interacting with each of the determined common genes

Meanwhile, a total of 13 targets, distributed among the compounds, were profoundly related to NSCLC thus, recognized as candidate targets. Inspection of the targeted genes (Fig. 3, Table 2) indicated that the genes AR, EGFR, and ESR-1 targets were the most enriched genes being common to compounds **6**, **4**, and **3**, respectively. This suggested their important role in the entire pathogenesis of NSCLC as well as the synergistic effect of these compounds in treating NSCLC, as they possibly act on some of the same biological processes or pathways. Several studies have documented the relationship between those genes and inflammation. For example, it was shown that male patients diagnosed with lung cancer exhibited greater survival when exposed to androgen pathway manipulation (APM) (Harlos et al. 2015). Also, cell proliferation, migration, invasion, and tumor formation were repressed by the interference of androgen receptor (AR) in NSCLC cell lines (Yeh et al. 2012). Tumor xenograft assay experiments confirmed that NSCLC tumorigenesis could be abolished by stimulating AR and epithelial-mesenchymal transition (EMT) mediation (Zhou et al. 2021). In the same context, drugs possessing dual action on VEGF-EGFR receptors have been approved and established for use in NSCLC (Le et al. 2021). Moreover, targeting FBXL2-Grp94 pathways might serve as a promising therapeutic strategy for tyrosine kinase inhibitors (TKI)-resistant NSCLC (Niu et al. 2021).

The importance of ESR-1 in improving the prognostic outcome of NSCLC was investigated using anti-hormonal drugs (Skjefstad et al. 2016; Enwere et al. 2020). Meanwhile, EGFR and ER were observed to cooperate in early activation of p42/p44 MAP kinase in NSCLC cells; hence, the synergistic use of antiestrogens with growth factor receptor antagonists could lead to the development of novel palliative strategies in NSCLC treatment.

Drawing support from target fishing, the aforementioned lung cancer targets were forwarded to KEGG functional enrichment analyses using the STRING database. KEGG analysis showed that the manifestation and progress of lung cancer were related to 39 NSCLC-related pathways, the most strikingly associated with NSCLC were pathways in cancer, proteoglycans in cancer, central carbon metabolism in cancer, EGFR tyrosine kinase inhibitor resistance, and endocrine resistance (Table 3).

**Table 3:** KEGG pathway analysis of potential target genes functions

#Term ID	Term description	Observed gene count	False discovery rate	Matching proteins in network (labels)
hsa05200	Pathways in cancer	12	5.71E − 16	PIK3CA, ERBB2, EGFR, PPARG, KIT, MAP2K1, TERT, MET, MTOR, AR, ALK, ESR1
hsa05205	Proteoglycans in cancer	8	1.19E − 11	PIK3CA, ERBB2, EGFR, MAP2K1, MET, MTOR, SRC, ESR1
hsa05230	Central carbon metabolism in cancer	7	2.83E − 12	PIK3CA, ERBB2, EGFR, KIT, MAP2K1, MET, MTOR
hsa01521	EGFR tyrosine kinase inhibitor resistance	7	4.25E − 12	PIK3CA, ERBB2, EGFR, MAP2K1, MET, MTOR, SRC
hsa01522	Endocrine resistance	7	1.19E − 11	PIK3CA, ERBB2, EGFR, MAP2K1, MTOR, SRC, ESR1
hsa05224	Breast cancer	7	1.30E − 10	PIK3CA, ERBB2, EGFR, KIT, MAP2K1, MTOR, ESR1
hsa05226	Gastric cancer	7	1.30E − 10	PIK3CA, ERBB2, EGFR, MAP2K1, TERT, MET, MTOR
hsa04151	PI3K-Akt signaling pathway	7	2.55E − 08	PIK3CA, ERBB2, EGFR, KIT, MAP2K1, MET, MTOR
hsa05223	Non-small cell lung cancer	6	1.67E − 10	PIK3CA, ERBB2, EGFR, MAP2K1, MET, ALK
hsa04012	ErbB signaling pathway	6	4.64E − 10	PIK3CA, ERBB2, EGFR, MAP2K1, MTOR, SRC
hsa05215	Prostate cancer	6	9.64E − 10	PIK3CA, ERBB2, EGFR, MAP2K1, MTOR, AR
hsa05206	MicroRNAs in cancer	6	1.69E − 08	PIK3CA, ERBB2, EGFR, MAP2K1, MET, MTOR
hsa05225	Hepatocellular carcinoma	6	1.69E − 08	PIK3CA, EGFR, MAP2K1, TERT, MET, MTOR
hsa04510	Focal adhesion	6	4.30E − 08	PIK3CA, ERBB2, EGFR, MAP2K1, MET, SRC
hsa04015	Rap1 signaling pathway	6	4.53E − 08	PIK3CA, EGFR, KIT, MAP2K1, MET, SRC
hsa05212	Pancreatic cancer	5	2.65E − 08	PIK3CA, ERBB2, EGFR, MAP2K1, MTOR
hsa05235	PD-L1 expression and PD-1 checkpoint pathway in cancer	5	5.35E − 08	PIK3CA, EGFR, MAP2K1, MTOR, ALK
hsa04066	HIF-1 signaling pathway	5	1.24E − 07	PIK3CA, ERBB2, EGFR, MAP2K1, MTOR
hsa04919	Thyroid hormone signaling pathway	5	2.05E − 07	PIK3CA, MAP2K1, MTOR, SRC, ESR1
hsa04915	Estrogen signaling pathway	5	3.18E − 07	PIK3CA, EGFR, MAP2K1, SRC, ESR1
hsa04072	Phospholipase D signaling pathway	5	4.94E − 07	PIK3CA, EGFR, KIT, MAP2K1, MTOR
hsa05163	Human cytomegalovirus infection	5	2.54E − 06	PIK3CA, EGFR, MAP2K1, MTOR, SRC
hsa04014	Ras signaling pathway	5	2.83E − 06	PIK3CA, EGFR, KIT, MAP2K1, MET
hsa04010	MAPK signaling pathway	5	8.62E − 06	ERBB2, EGFR, KIT, MAP2K1, MET
hsa05165	Human papillomavirus infection	5	1.46E − 05	PIK3CA, EGFR, MAP2K1, TERT, MTOR
hsa05219	Bladder cancer	4	2.88E − 07	ERBB2, EGFR, MAP2K1, SRC
hsa05213	Endometrial cancer	4	8.72E − 07	PIK3CA, ERBB2, EGFR, MAP2K1
hsa05221	Acute myeloid leukemia	4	1.46E − 06	PIK3CA, KIT, MAP2K1, MTOR
hsa04520	Adherens junction	4	1.49E − 06	ERBB2, EGFR, MET, SRC
hsa04917	Prolactin signaling pathway	4	1.60E − 06	PIK3CA, MAP2K1, SRC, ESR1
hsa05214	Glioma	4	1.82E − 06	PIK3CA, EGFR, MAP2K1, MTOR
hsa05218	Melanoma	4	1.82E − 06	PIK3CA, EGFR, MAP2K1, MET
hsa05210	Colorectal cancer	4	2.70E − 06	PIK3CA, EGFR, MAP2K1, MTOR
hsa05231	Choline metabolism in cancer	4	4.64E − 06	PIK3CA, EGFR, MAP2K1, MTOR
hsa04926	Relaxin signaling pathway	4	1.33E − 05	PIK3CA, EGFR, MAP2K1, SRC
hsa05167	Kaposi sarcoma-associated herpesvirus infection	4	5.47E − 05	PIK3CA, MAP2K1, MTOR, SRC
hsa04810	Regulation of actin cytoskeleton	4	7.97E − 05	PIK3CA, EGFR, MAP2K1, SRC
hsa05131	Shigellosis	4	9.15E − 05	PIK3CA, EGFR, MTOR, SRC

**Fig. 4:**
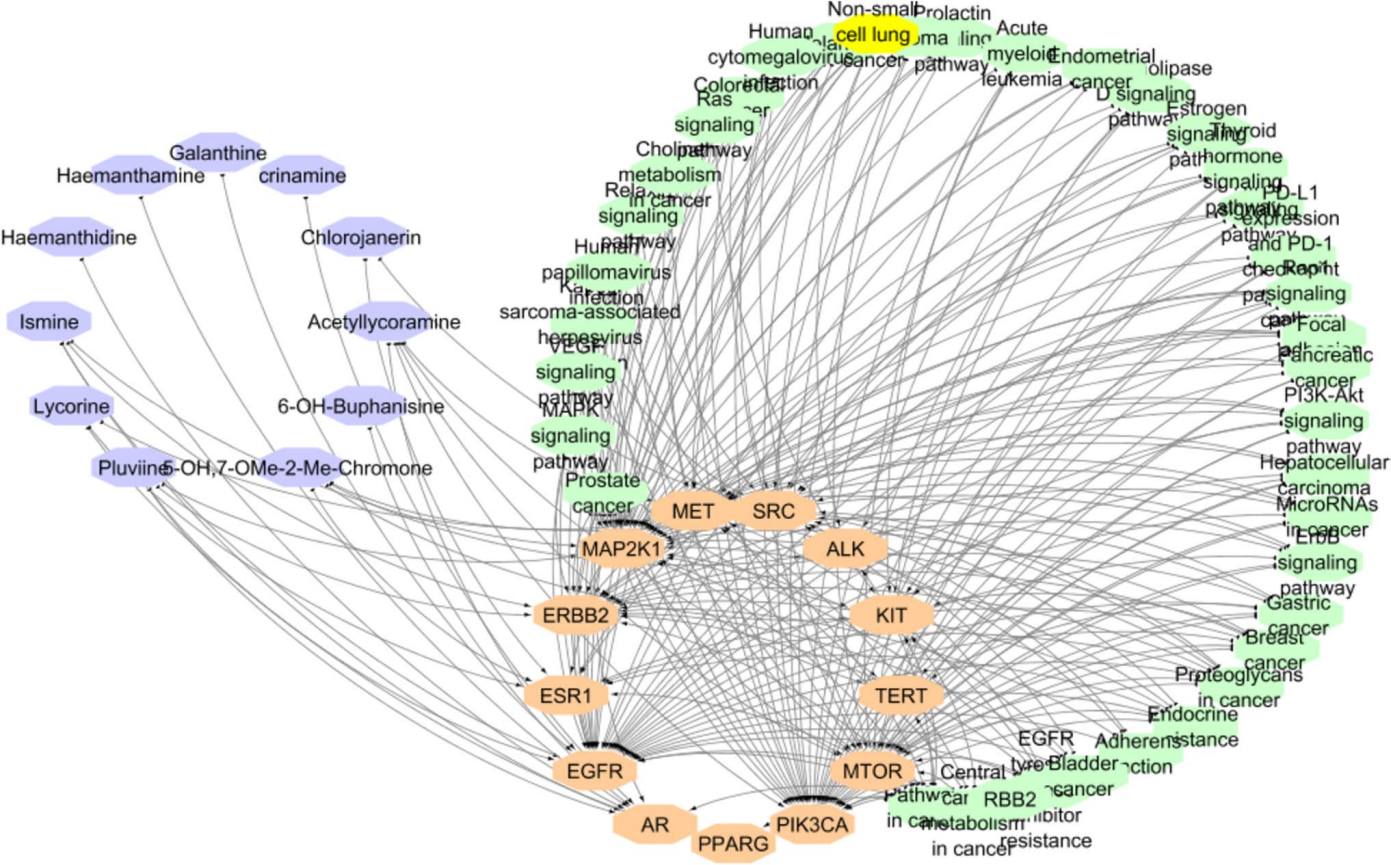
Network of compound-target gene-pathway interactions for the top-scored isolates with 13 target proteins and 39 pathways presented by violet, orange, and green heptagon nodes, respectively

Interactions between the top-scored compounds with the proteins and pathways involved in NSCLC were unveiled via constructing a constituent-target-pathway network (Fig. 4) including 48 nodes (11 compound nodes, 13 target nodes, 37 pathway nodes) and 198 edges. These data revealed the poly-pharmacology and multi-target properties of these compounds.

**Fig. 5:**
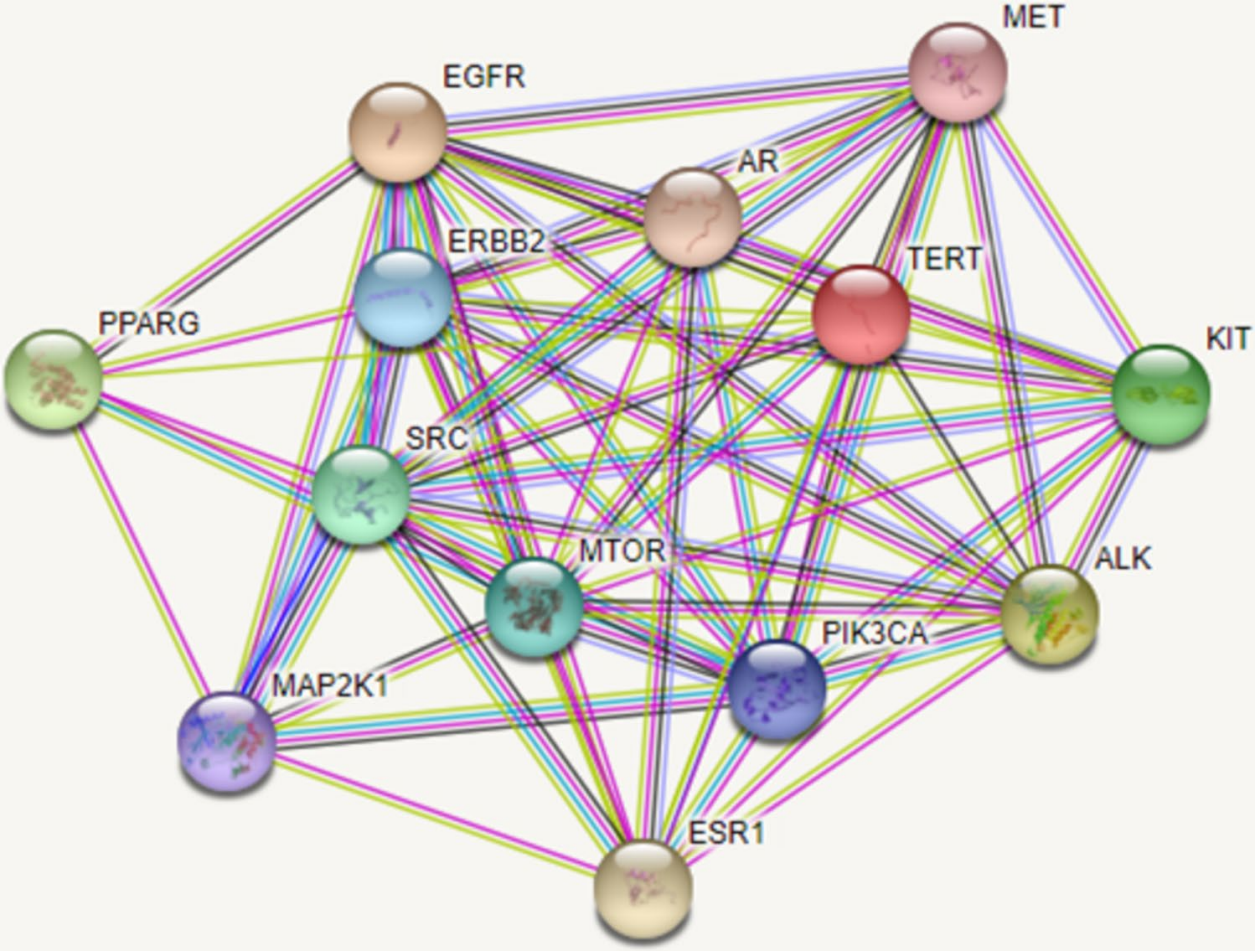
Protein–protein interaction (PPI) network of the putative NSCLC-related targets

Protein–protein interactions were examined using the STRING database and then visualized through P-P network analysis. From this network, strong correlations between the identified potential NSCLC target proteins were spotted, suggesting that they probably regulate the functions of each other (Fig. 5).

#### Gene ontology enrichment analysis

**Fig. 6:**
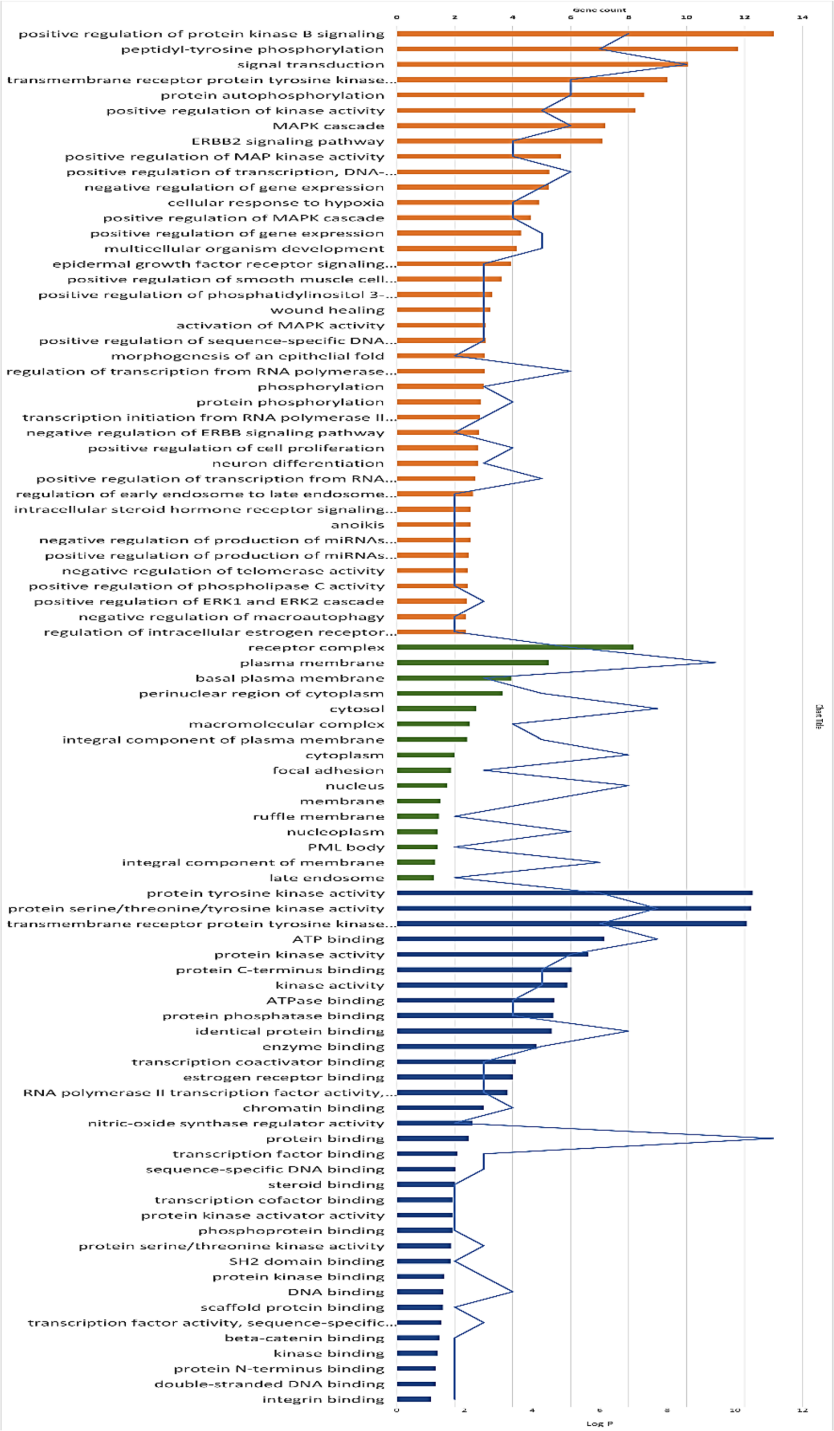
Gene ontology analysis of the putative targets of NSCLC by the DAVID database. BP, MF, and CC terms are represented by orange, green, and blue bars, respectively. The significance of enrichment is indicated by log *p*-value with bar charts. The blue line represents the number of genes enriched by each term

Common genes were subjected to GO enrichment analysis using the DAVID database to illustrate the underlying mechanism of the top-scored isolates in mitigating NSCLC using biological process (BP), cellular component (CC), and molecular function (MF) terms. For instance, BP describes gene participation from the cellular to systemic level. CC presents the protein location inside/outside the cell, while MF demonstrates the protein functions at the molecular level (Lagunin et al. 2020). It is considered a statistically-based approach intended for the determination of over-expressed groups of proteins that may have an association with a certain disease (Lagunin et al. 2020). According to preliminary results (Fig. 6), a total of 40 BP terms were selected, the most related to NSCLC included positive regulation of protein kinase-B signaling, peptidyl-tyrosine phosphorylation, signal transduction, transmembrane receptor protein tyrosine kinase signaling pathway, and protein autophosphorylation. Concurrently, five key targets were primarily distributed in cellular components such as receptor complex, plasma membrane, basal plasma membrane, perinuclear region of cytoplasm, and cytosol. According to enrichment analysis of molecular functions, the targets were mainly involved in protein tyrosine kinase activity, protein serine/threonine/tyrosine kinase activity, transmembrane receptor protein, tyrosine kinase activity, ATP binding, and protein kinase activity.

**Fig. 7:**
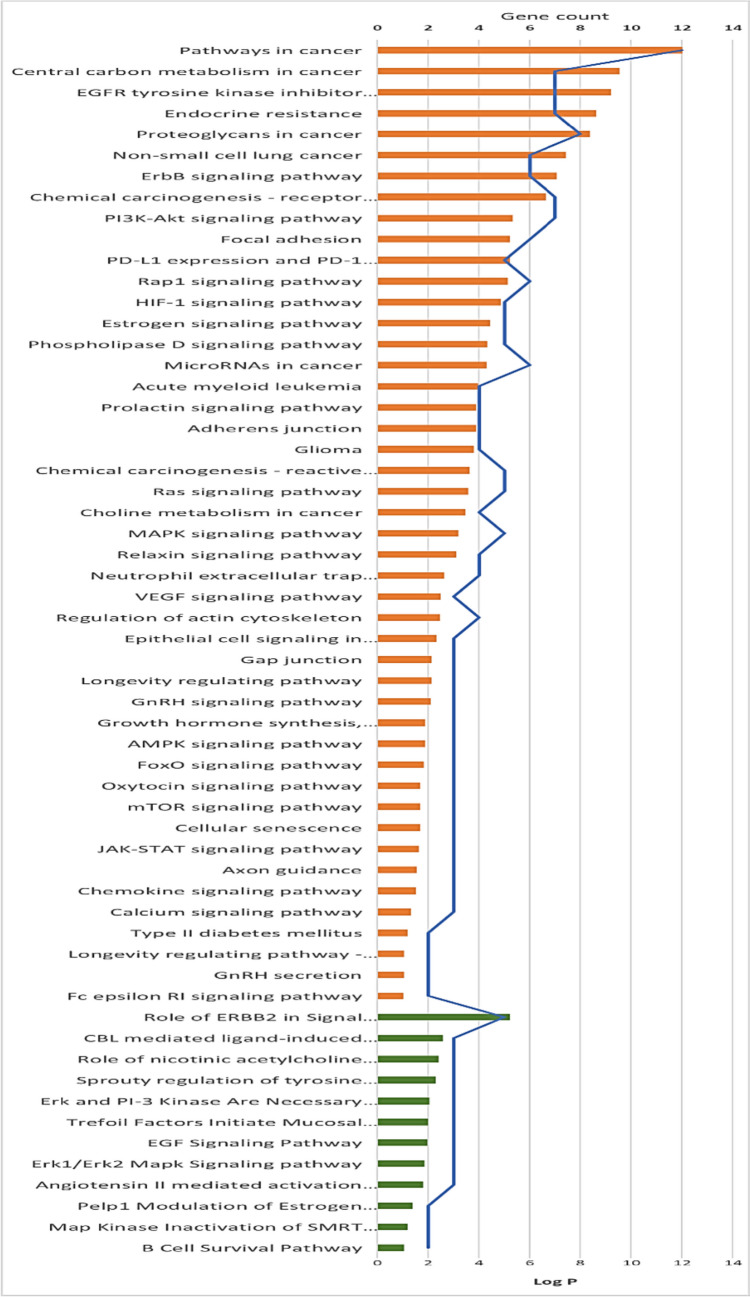
Major KEGG (orange) and BIOCARTA (green) pathways clusters generated from the DAVID database. The significance of enrichment is indicated by log *p*-value with bar charts. Blue lines represent the number of genes enriched by each term

Pathways functional enrichment analysis (Fig. 7) allowed for the determination of the signaling pathways and functions of identified target genes where 46 KEGG pathways were recognized (*p* < 0.05). Pathways in cancer were the most enriched with the highest number of observed genes and lowest false discovery rate followed by central carbon metabolism in cancer, EGFR tyrosine kinase inhibitor resistance, and endocrine resistance. Further, NSCLC was observed to be strongly modulated by 12 BIOCARTA pathways, the most positively associated of which were ERBB2 in signal transduction and oncology and CBL-mediated ligand-induced downregulation of EGF receptors.

Based on the above network pharmacology analysis results, the four top-scored compounds named acetyllycoramine (**19**), ismine (**16**), pluviine (**10**), and 5-hydroxy,7-methoxy-2-methylchromone (**11**) were subjected to molecular docking studies aiming to afford deepened overview of their multi-targeted molecular mechanisms.

### Molecular docking and dynamics studies

Firstly, we made use of the RMSD value for validation of the docking protocol which was obtained through the use of the ligand co-crystallized with each crystal structure (PDB ID: 2PIW, 1M17, and 6CBZ). As highlighted in Table 4, for the three protein target crystal structures, the RMSD value was less than 1 A, which reflects high docking accuracy (Dawood et al. 2023; Ibrahim et al. 2021).

**Table 4:** RMSD and XP-G scores of the top four scoring compounds and erlotinib docked into AR, EGFR and ESR-1 active sites (expressed in kcal/mol)

Compound	Proteins		
	AR	EGFR	ESR-1
*RMSD (co-crystallized ligand)	0.445	0.785	0.635
Ismine (**16**)	− 9.204	− 7.529	− 9.104
5-hydroxy-7-methoxy-2-methylchromone (**11**)	− 7.316	− 6.111	− 6.978
Pluviine (**10**)	− 6.791	− 4.654	− 6.485
Acetyllycoramine (**19**)	− 6.094	− 3.984	− 5.689
**Erlotinib	− 3.800	− 9.034	− 9.060

^*^RMSD values were calculated for each enzyme using the enzyme’s crystalline structure and its respective co-crystallized ligand

^**^Erlotinib is used as positive control

**Fig. 8:**
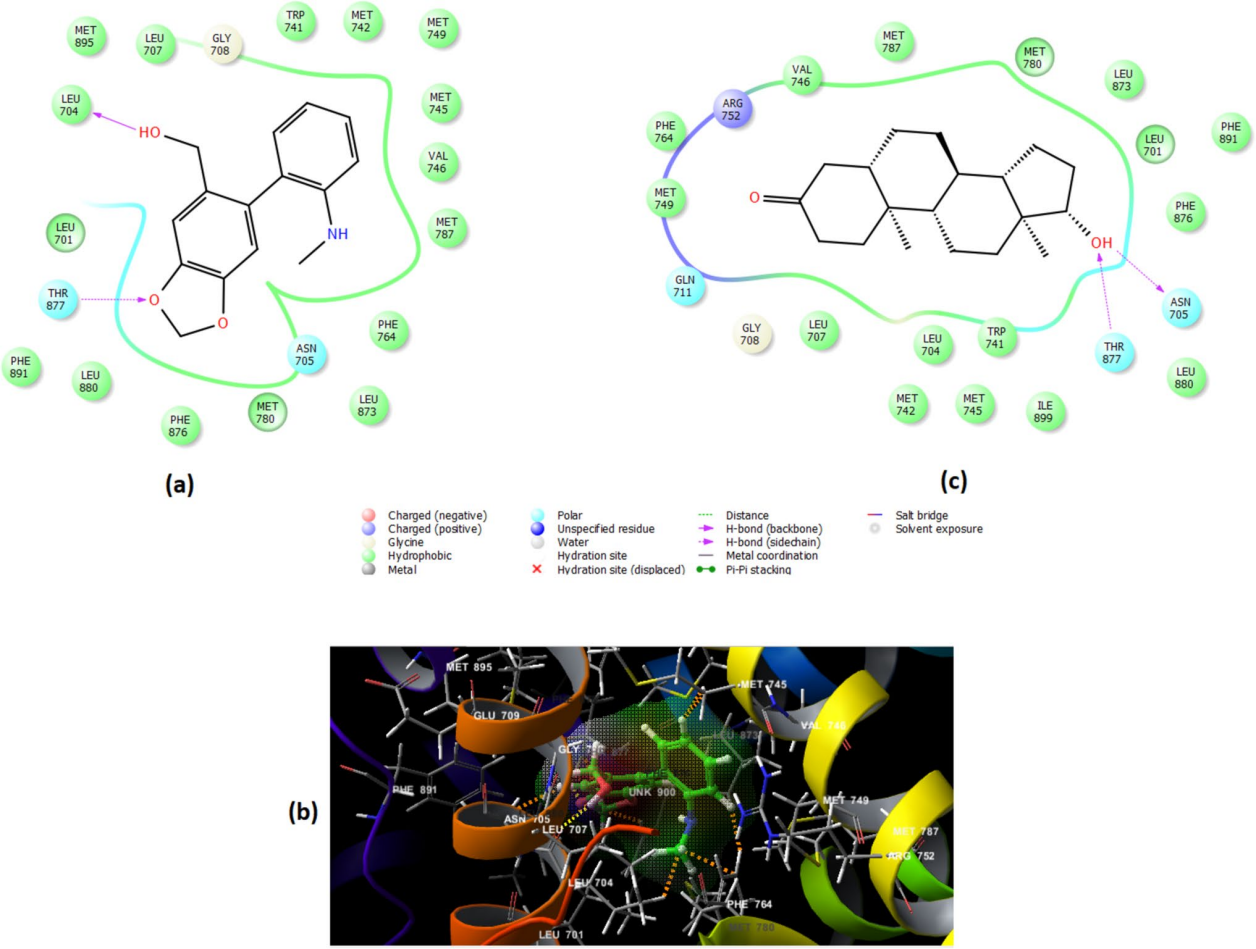
**a** 2D and **b** 3D ligand interaction diagrams for docking poses of ismine in the active site of AR crystalline structure (PDB ID: 2PIW) together with **c** 2D interaction diagram of the co-crystallized ligand dihydrotestosterone

**Fig. 9:**
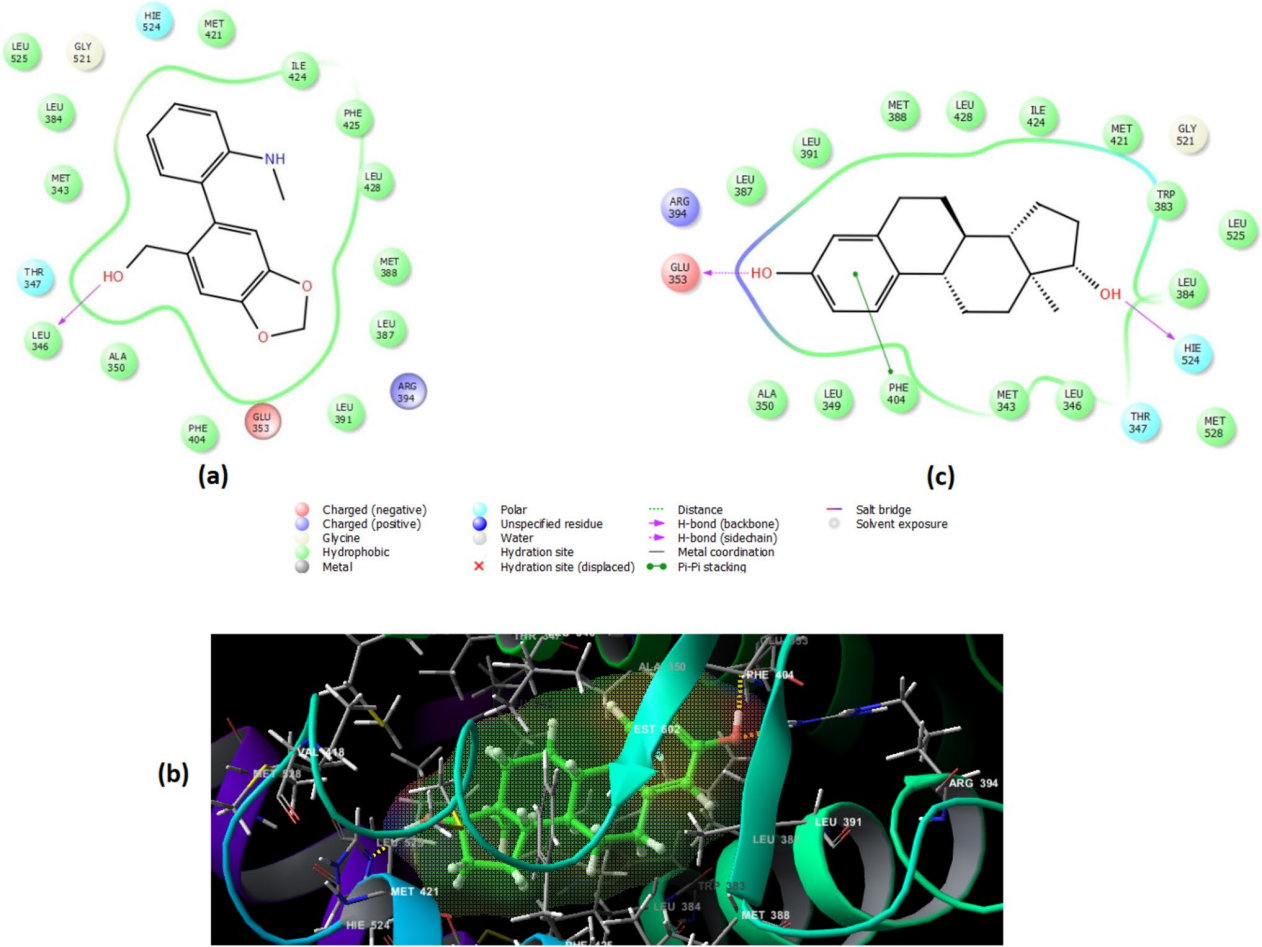
**a** 2D and **b** 3D ligand interaction diagrams for docking poses of ismine in the active site of ESR-1 crystalline structure (PDB ID: 6CBZ) together with (**c**) 2D interaction diagram of the co-crystallized ligand estradiol

**Fig. 10:**
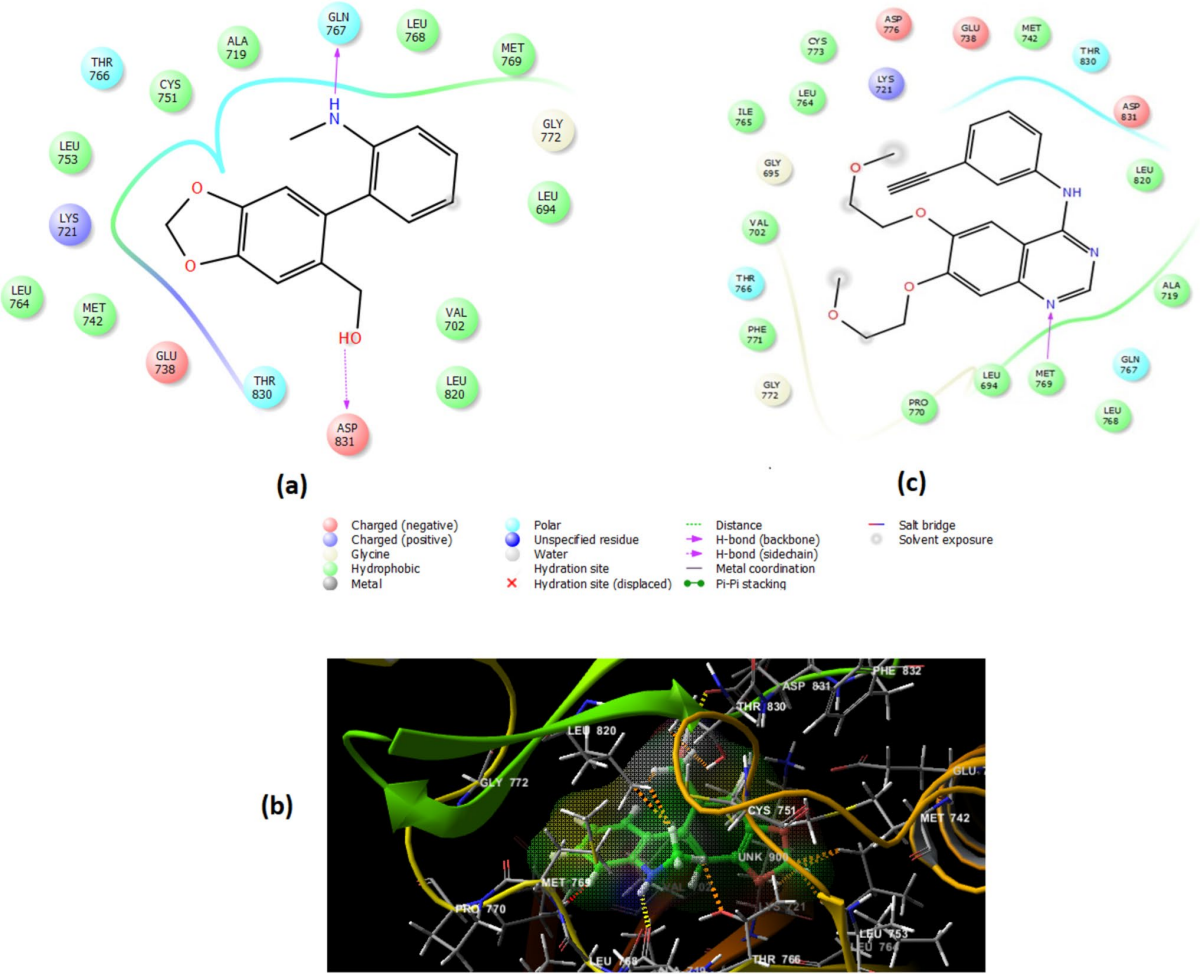
**a** 2D and **b** 3D ligand interaction diagrams for docking poses of ismine in the active site of EGFR crystalline structure (PDB ID: 1m17) together with **c** 2D interaction diagram of the co-crystallized ligand erlotinib

To study the molecular interactions between the top four hit constituents and the three most enriched target genes (AR, EGFR, and ESR-1), molecular docking studies were conducted, and their 2D and 3D interaction patterns are shown in Figs. 8, 9, and 10. Among all of the tested ligands analyzed, ismine (**16**) performed best in multi-regimen inhibition of lung cancer cell growth. It showed efficient binding, with all three receptor proteins having the most negative binding energies among all ligand–receptor interactions, followed by 5-hydroxy-7-methoxy-2-methylchromone (**11**), pluviine (**10**), and finally acetyllycoramine (**19**) (Table 4). Energetically, ismine (**16**) and AR exhibited the most favored interaction, with a binding energy of − 9.304 kcal/mol. During this interaction, two hydrogen bonds were formed with the amino acids Leu704 and THR877, 16 hydrophobic interactions with backbone amino acid residues in addition to two polar interactions with ASN705 and Leu704 (Fig. 8a and b). Additionally, Fig. 8c represents the 2D interaction diagram of the co-crystallized ligand (dihydrotestosterone).

Concerning docking into the ESR-1 pocket, results showed that ismine (**16**) displayed weaker binding affinity (− 9.104 kcal/mol) where it shared one hydrogen bonding with Leu346, 13 hydrophobic interactions, two polar interactions with THR347 and HIE524, as well as a positive- and negative-charged interactions with ARG394 and GLU353, respectively (Fig. 9a and b). Moreover, Fig. 9c explains the 2D interaction diagram of the co-crystallized ligand, estradiol.

As shown in the 2D interaction diagram of ismine (**16**) with EGFR (Fig. 10a and b), it extended into the active site via two hydrogen bondings with the amino acids GLN767 and ASP831, ten hydrophobic interactions, three polar interactions with GLN767, THR766, and THR830, and two negative interactions with ASP831 and GLU738 beside another positive interaction with LYS721. In terms of docking scores, ismine (**16**) displayed the weakest binding affinity, scoring (− 7.529 kcal/mol), when compared to the other receptor proteins. Finally, Fig. 10c shows a 2D interaction diagram of the co-crystallized ligand; erlotinib.

It can also be noted that ismine (**16**) showed similar interaction patterns to that of the co-crystalized ligands dihydrotestosterone, estradiol, and erlotinib inside the pockets of AR, ESR-1, and EGFR proteins, respectively (Figs. 8c, 9c, and 10c). This similarity in interactions between ismine and the correct amino acid residues of the three protein targets confirmed that ismine was successfully docked into the correct active sites. Importantly, the presence of ismine in the proper pockets of the three enzymes represents a high potential for this ligand to exert its action with the same underlying mechanism of action. Eventually, this triple action of ismine on the three main top targets of NSCLC urged it as a pharmacologically active candidate for this disease.

For the sake of confirmation of the potential activity of ismine, its docking scores and interactions were compared to erlotinib. Erlotinib is an orally administered small molecular inhibitor of lung cancerous cell line. It causes G0/G1 cell cycle arrest and inhibits cancer cell proliferation. In sensitive cells, erlotinib causes tumor cell apoptosis (Piperdi and Perez-Soler 2012). After performing molecular docking analysis of erlotinib on the three targets—AR, ESR-1, and EGFR—it was astonishing to observe that ismine possessed more favorable docking scores with both AR and ESR-1 compared to erlotinib which possessed docking scores of − 3.800 and − 9.060 kcal/mol, respectively. Meanwhile, erlotinib exhibited a slightly better score (− 9.034 kcal/mol) on EGFR (Table 4). These results raised ismine as a promising candidate for the treatment of NSCLC. The 2D interaction diagrams of erlotinib with AR, ESR-1, and EGFR are depicted in (Fig. S2) in the supplementary materials.

While the number of interactions between the amino acid residues and a compound does not necessarily reflect its true pharmacological activity as the type, the strength of interactions and their inter-site distances play a crucial role in stabilizing the ligand-protein complex which in turn affects its binding affinity (Khaerunnisa, 2020). That could explain the lower docking score of ismine (**16**) when bound to EFGR compared to ESR-1 despite possessing a higher number of interactions.

**Fig. 11:**
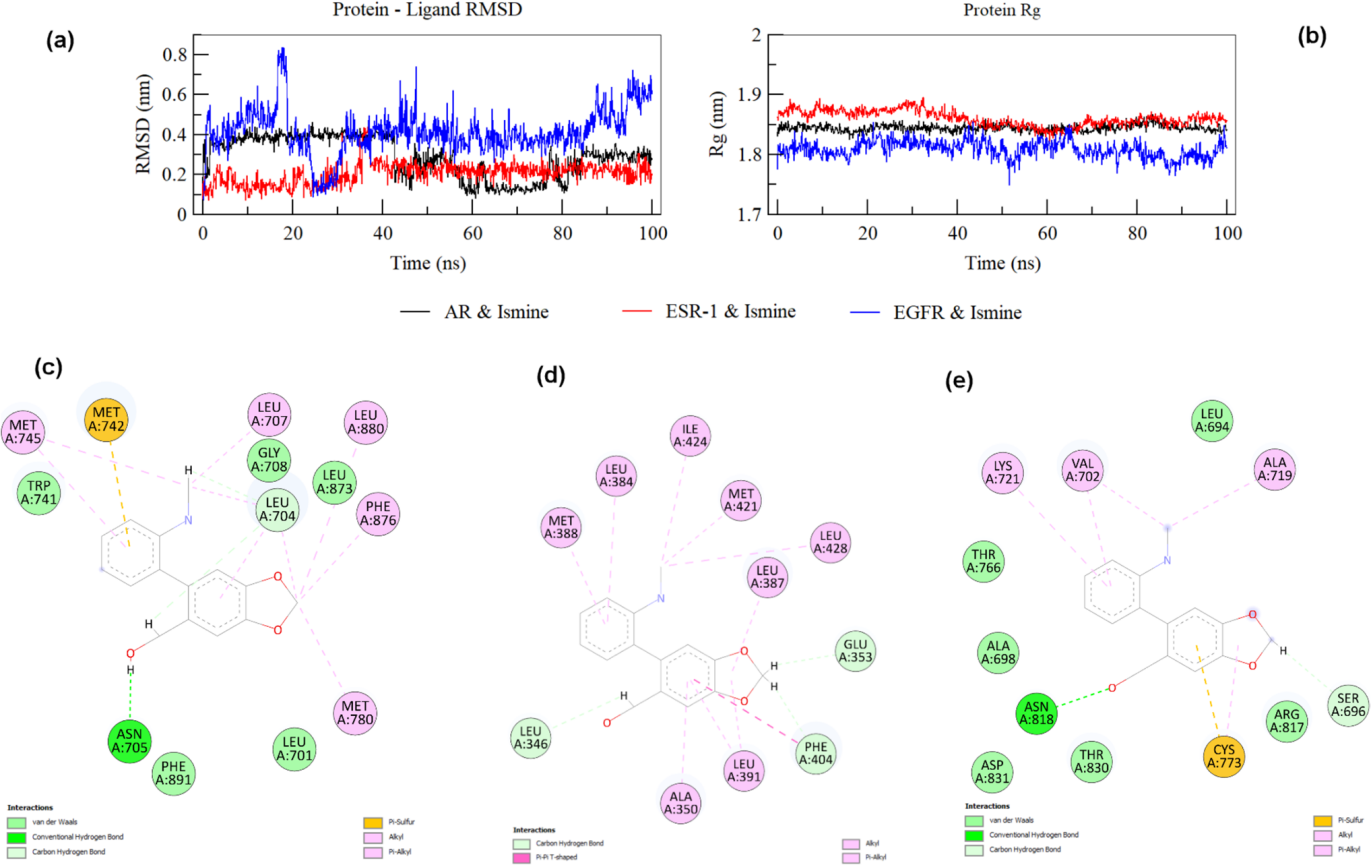
**a** Protein–ligand RMSD analysis over 100 ns for AR, ESR-1, and EGFR with ismine, showing stability and fluctuations. **b** Protein Rg analysis indicating compactness during the simulation. **c** Interaction map of AR-ismine at 100 ns, showing stable key interactions. **d** Interaction map of ESR-1-ismine at 100 ns, with consistent interactions and enhanced contacts. **e** Interaction map of EGFR-ismine at 100 ns, displaying maintained interactions with minor flexibility

To validate the docking results and explore the dynamic stability of ismine in complex with AR (PDB ID, 2PIW), ESR-1 (PDB ID, 6CBZ), and EGFR (PDB ID, 1M17), 100-ns molecular dynamics (MD) simulations were conducted. The MD trajectories were analyzed for stability, key interactions, and conformational changes over time (Fig. 11a and b).

The MD simulation of the AR-ismine complex demonstrated high stability over 100 ns, with RMSD values stabilizing at ~ 0.2 to 0.3 nm, indicating minimal conformational changes, and the radius of gyration (Rg) values consistently around 1.75–1.8 nm, reflecting a compact structure. Key hydrogen bonds with LEU704 and THR877 observed in the docking pose were preserved throughout the simulation, along with polar interactions with ASN705. Hydrophobic contacts with residues such as PHE876, MET742, and LEU707 remained consistent for time (Fig. 11c). The 100-ns pose showed slight adjustments that optimized hydrogen bonding and hydrophobic interactions, aligning closely with the docking pose and confirming AR as the most stable binding target for ismine (Video S1).

In the ESR-1-ismine complex, RMSD values were slightly higher (~ 0.3 to 0.4 nm), reflecting moderate stability, and the Rg values remained consistent at ~ 1.8 to 1.85 nm, indicating a stable but less compact structure compared to AR (Fig. 11B). Hydrogen bonding with LEU346 was retained, but the bond with GLU353, observed in the docking pose, was intermittently lost. Hydrophobic interactions with residues such as LEU387, LEU391, and MET421 remained stable, while additional contacts with deeper residues like LEU428 emerged over time (Fig. 11d). The 100-ns pose exhibited some deviation from the docking pose, with slight rearrangements enhancing hydrophobic contacts and compensating for the loss of some polar interactions (Video S2).

The EGFR-ismine complex showed the highest RMSD (~ 0.4 to 0.6 nm), indicating lower stability and greater flexibility, with Rg values fluctuating between 1.85 and 1.9 nm, reflecting a more dynamic and less compact structure. While the hydrogen bond with ASN818 was maintained, the bond with SER696 was lost early in the trajectory. Hydrophobic contacts with residues such as ALA719, VAL702, and CYS773 persisted but weakened due to the ligand’s flexibility (Fig. 11e). The 100-ns pose deviated significantly from the docking pose, with reduced hydrogen bonding and weaker hydrophobic interactions, confirming the relatively lower stability of ismine in the EGFR binding pocket compared to AR and ESR-1 (Video S3).

The MMPBSA results align with the MD simulation findings (Table S1), highlighting AR as the most stable and favorable binding target for ismine, with a binding energy of − 90.6 kJ/mol, driven by strong van der Waals and electrostatic interactions. The ESR-1 complex showed moderate stability, with a binding energy of −83.6 kJ/mol, reflecting a balance between hydrophobic and polar contributions despite the loss of some hydrogen bonds during the simulation. In contrast, the EGFR complex exhibited the weakest binding energy (− 39.5 kJ/mol), attributed to weaker van der Waals forces and higher polar solvation energy, consistent with its higher RMSD, fluctuating Rg, and dynamic behavior. These results confirm AR as the most suitable target for ismine, followed by ESR-1, with EGFR showing limited stability.

### In silico ADMET study

QikProp was used to determine the ADMET properties of the top four hit compounds (**10**, **11**, **16**, **19**). It is a useful tool that can provide information regarding the drug-likeness of the compound, its oral absorption, permeability to barriers, metabolic reactions, and toxicity. This valuable information can give a guide to possible chemical modifications to improve its activity (Divyashri et al. 2021).

The main objective of this study was to determine the drug-likeness properties of the tested compounds. This was attained by checking whether they obeyed Lipinski’s rule of five which includes some descriptors with specific ranges named Mol_MW < 500, Qplogpo/w < 5, Donorhb ≤ 5, and Accpthb ≤ 10 (Saini et al. 2022). Analyzing Lipinski’s rule of five for the four compounds (Table 5) showed that all of them exhibited no violations for all these descriptors implying their high drug-likeness potentials. Oral absorption is assessed by some parameters such as the predicted aqueous solubility (QPlogS, − 6.0 to 0.5), the predicted percentage of human oral absorption (25–80%), and obeying Jorgensen’s rule of three. A compound complying with all or some of the rules (QPlogS >  − 5.7, Caco2 > 22 nm/s, and # primary metabolites < 7) is considered to be orally available with probable high therapeutic activity as it possessed good aqueous solubility (represented by QPlogS) and satisfying hydrophobicity (represented by Caco2) so that it can easily penetrate the gut walls (James et al. 2023). As depicted in Table 5, all the tested compounds exhibited satisfying values for QPlogS, great percentages of human oral absorption, and successfully obeyed the rule except for pluviine (**10**) which exceeded the maximum allowed number of metabolic reactions (#metb = 8), thus alerting for the need to apply some chemical and structural modifications to enhance its oral bioavailability.

**Table 5:** In silico ADMET profiles of the top four hit compounds

	mol MW	donorHB	accptHB	QPlogPo/w	QPlogS	QPlogHERG	QPPCaco	#metab	QPlogKhsa	Percent human oral absorption	Rule of five	Rule of three
Acetyllycoramine (**19**)	331.411	0	5.5	2.585	− 2.736	− 4.912	608.301	3	0.101	91.91	0	0
5-hydroxy-7-methoxy-2-methyl-chromone (**11**)	206.198	0	3	1.74	− 2.22	− 3.936	1239.99	3	− 0.316	92.503	0	0
Ismine (**16**)	257.288	2	4.2	2.363	− 2.564	− 4.034	3038.956	4	− 0.146	100	0	0
Pluviine (**10**)	287.358	1	5.2	2.332	− 2.538	− 4.921	1014.347	8	0.065	94.405	0	1

As well known, the binding of the drugs to plasma proteins reduces the concentration of the drug in the bloodstream, thus, abolishing its efficiency. The drug binding to the plasma protein albumin can be predicted by the parameter (QPlogKhsa) with a recommended range of − 1.5 to 1.5 (Sharma et al. 2024). All compounds lay in the recommended range (Table 5), suggesting their free blood circulation and their good availability to the target site.

The human Ether-a-go-go-related gene (hERG) encodes the pore-forming subunit of the rapidly activating delayed rectifier potassium channel (I_Kr_), which is important for cardiac electrical activity and heart beating. Dysfunction of hERG causes long QT syndrome and sudden death, which occur in patients with cardiac ischemia (Omoboyowa 2022). The predicted IC_50_ of drugs causing channel inhibition by 50% can be estimated using QPlogHERG. All the phytochemicals showed predicted IC_50_ value above the critical recommended value (− 5) indicating their low cardiotoxic effects.

## Conclusion

In summary, 21 alkaloidal and non-alkaloidal compounds were isolated from *C. bulbispermum*, *P. maritimum* and *H. vittatum* (Amaryllidaceae), and *C. scoporia* (Asteraceae) perennial plants of valuable medicinal importance. Among these, four compounds, crinamine (**17**), lycorine (**1**), hemanthidine (**5**), and haemanthamine (**6**), revealed profound in vitro cytotoxic activity (potency) towards NSCLC cells (greater than 100 units/µg). Interestingly, compounds **5**, **6**, and **17** belong to the haemanthamine-type alkaloids, while one belongs to the lycorine-type alkaloids. Further, one compound, pluviine, demonstrated solid tumor selectivity by the zone assay. These five compounds deserve consideration for further anticancer development. To our knowledge, this is the first report investigating their cytotoxic activity to that type of cancer cells. The study in hand has provided for the first time a comprehensive understanding of the suggested anti-NSCLC mechanisms for these plants based on the research strategy of network pharmacology followed by molecular docking studies. It was clarified that the core components namely ismine (**16**), 5-hydroxy-7-methoxy-2-methylchromone (**11**), pluviine (**10**), and acetyllycoramine (**19**) have multi-target mechanisms in mitigating lung cancer through the inhibition of AR, EGFR and ESR-1 proteins with involvement of 39 putative pathways. In silico ADMET study conducted on these compounds confirmed their excellent drug-likeness properties, oral bioavailability, and safety profiles, highlighting the need for some structural modifications to pluviine (10) to enhance its oral bioavailability. These results supported that these plants could serve as effective anti-tumor candidates believing that they may be useful for fostering innovative research of new drugs against NSCLC. However, the abovementioned in vitro cytotoxic activity results, potential target points, and mechanisms of action as well as the in silico pharmacokinetic profiles of compounds need to be extensively verified and augmented by more sophisticated pre-clinical and clinical research to afford a more scientific basis for the development and utilization of these plants and/or their secondary metabolites in treatment of NSCLC.

## Supplementary Information

Below is the link to the electronic supplementary material.

Supplementary file1 (DOCX 3356 KB)

Supplementary file2 (PPTX 86312 KB)

## Data Availability

All source data for this work (or generated in this study) are available upon reasonable request.
