# Supplementary material for: Molecular mechanisms of phytoconstituents from selected Egyptian plants against non-small cell lung cancer using integrated in vitro network pharmacology and molecular docking approach
Source: Naunyn Schmiedebergs Arch Pharmacol. 2025 Jan 31;398(7):9061–82. doi: 10.1007/s00210-025-03834-4 (PMC12263816; doi:10.1007/s00210-025-03834-4)
Supplement: Supplementary file 1 — Supplementary file1 (DOCX 3356 KB) [file 210_2025_3834_MOESM1_ESM.docx]

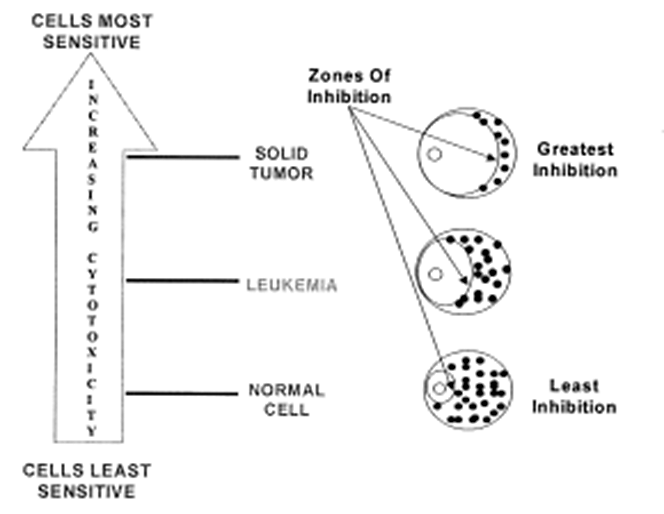


**Figure S1: The principle of disc- diffusion assay**


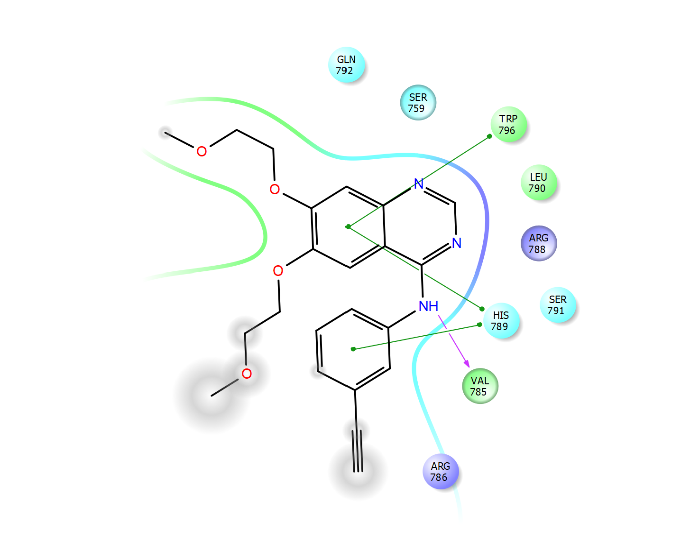

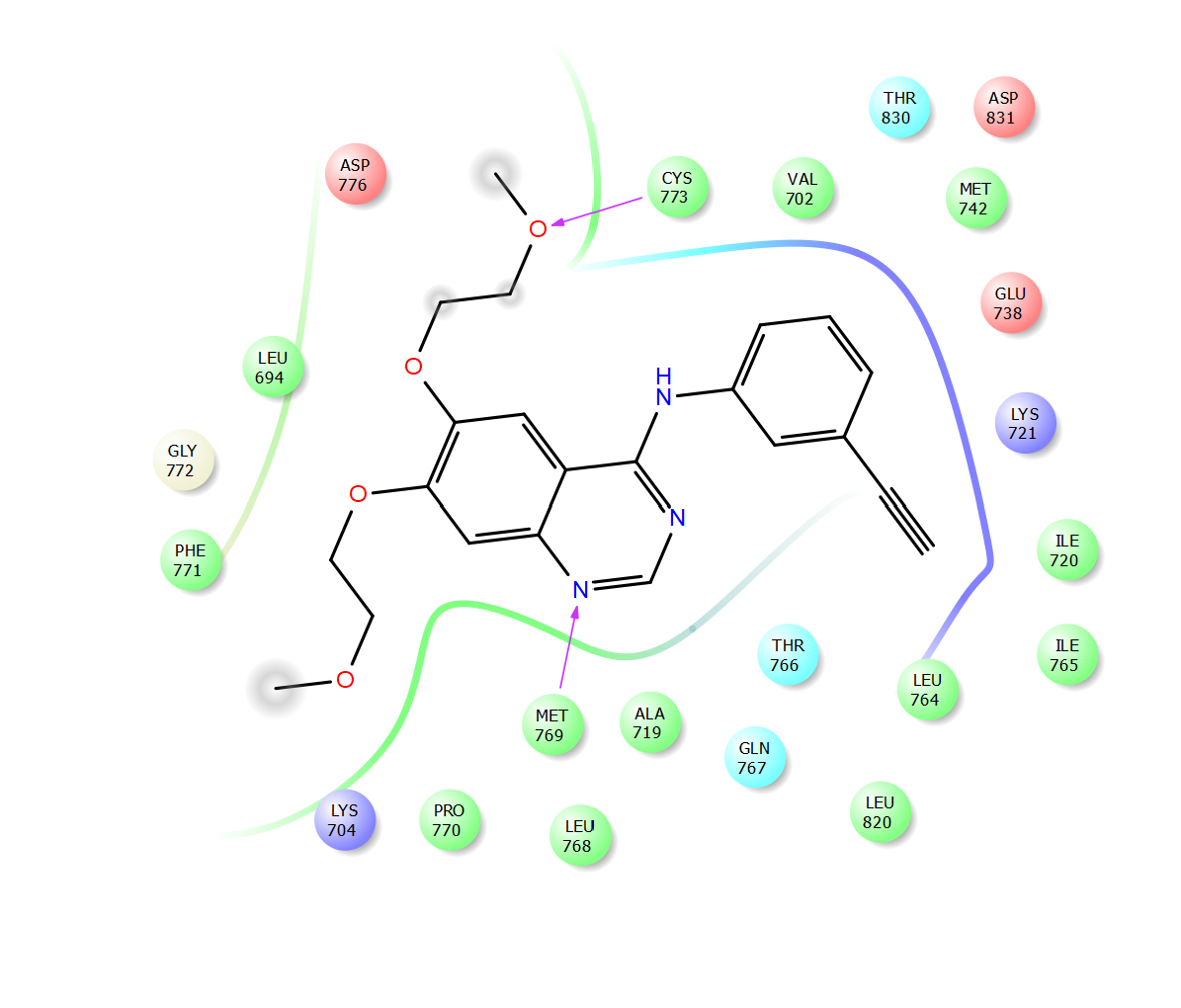

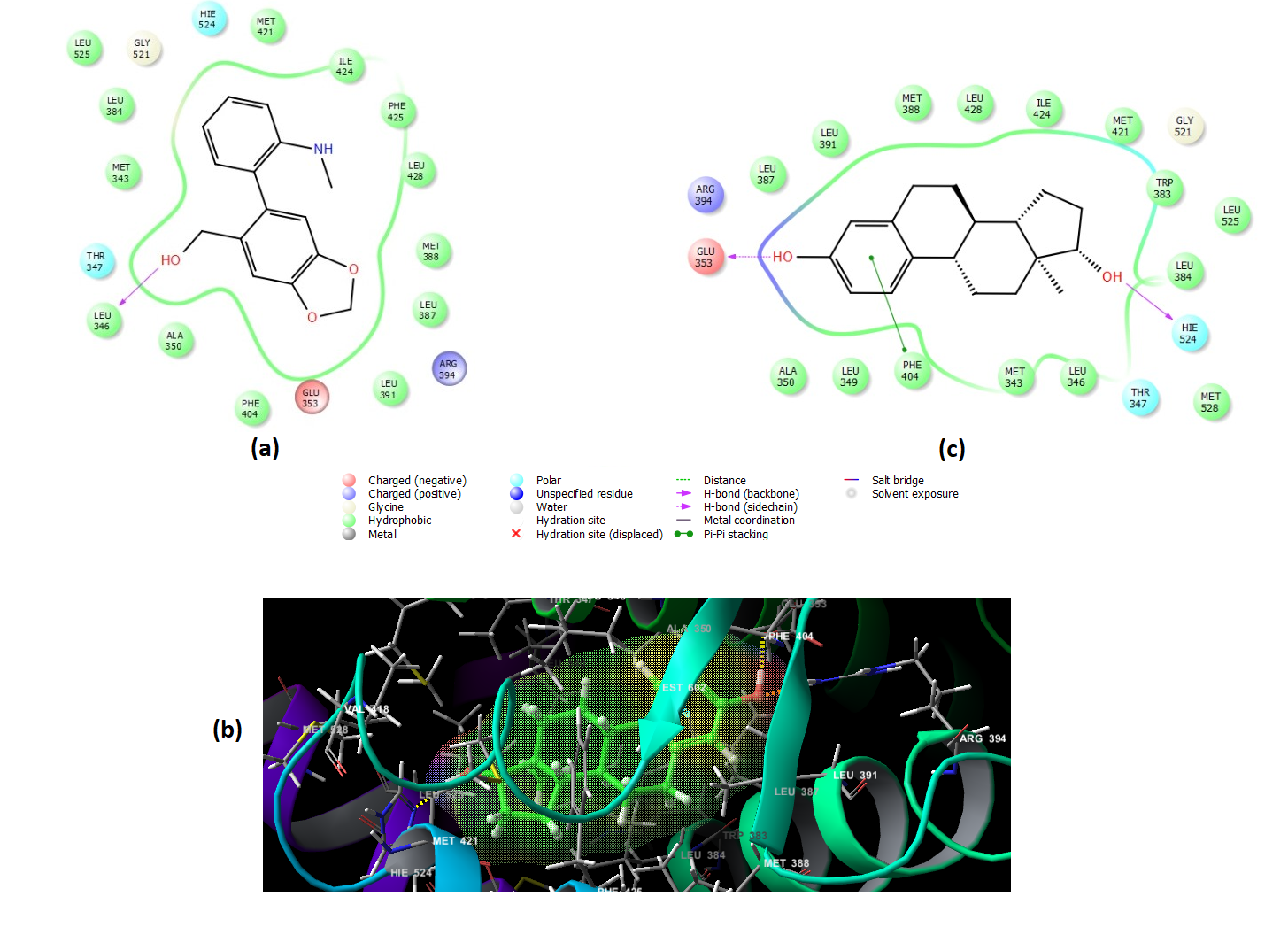

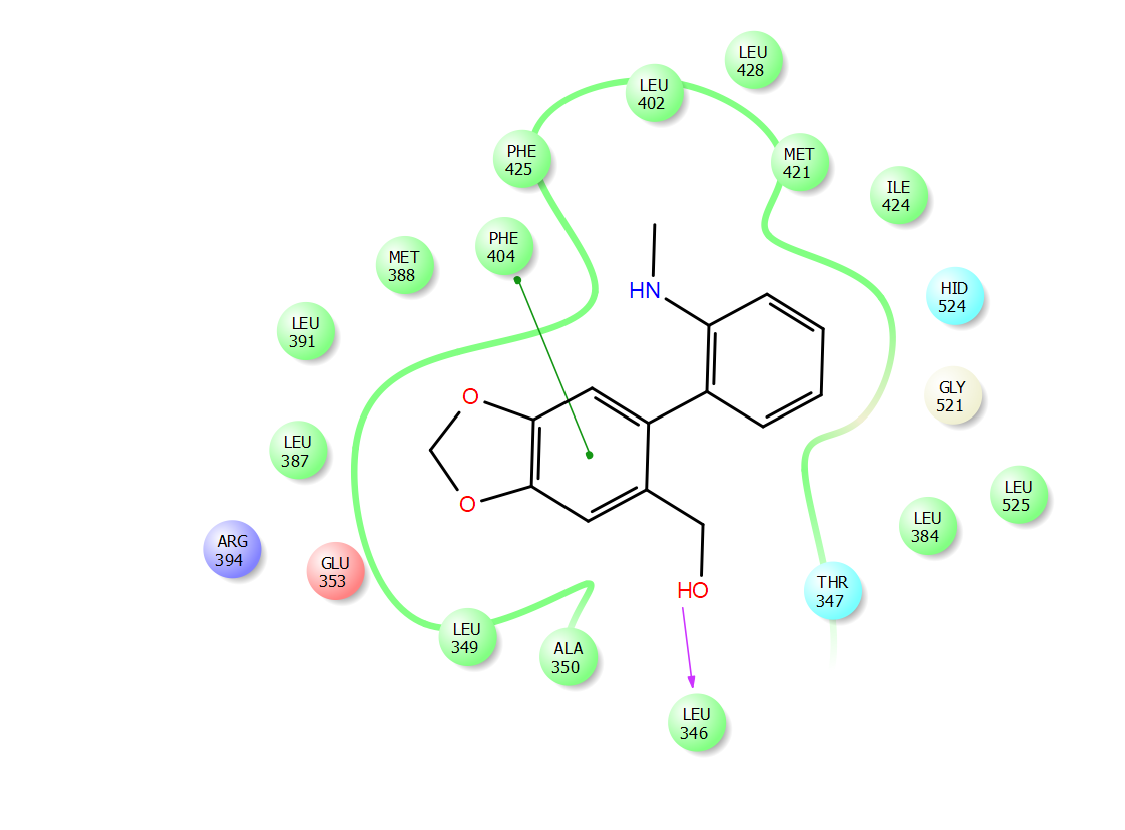


**(a)**

**(b)**

**(c)**

**Figure S2: 2D-interaction diagrams of erlotinib with AR (a), ESR-1 (b) and EGFR (c).**

**Table S1.** MMPBSA energy components for AR, ESR-1, and EGFR complexes with ismine calculated between 80 ns and 100 ns, highlighting van der Waals, polar solvation, SASA, and binding energies.

|  | van der Waal energy (kJ/mol) | Polar solvation energy (kJ/mol) | SASA energy (kJ/mol) | Binding energy (kJ/mol) |
| --- | --- | --- | --- | --- |
| AR | -147.1 | 93.0 | -15.8 | **-90.6** |
| ESR-1 | -150.9 | 101.0 | -15.7 | **-83.6** |
| EGFR | -114.2 | 113.9 | -15.0 | **-39.5** |
