# Supplementary material for: Molecular mechanisms of phytoconstituents from selected Egyptian plants against non-small cell lung cancer using integrated in vitro network pharmacology and molecular docking approach
Source: Naunyn Schmiedebergs Arch Pharmacol. 2025 Jan 31;398(7):9061–82. doi: 10.1007/s00210-025-03834-4 (PMC12263816; doi:10.1007/s00210-025-03834-4)
Supplement: Supplementary file 2 — Supplementary file2 (PPTX 86312 KB) [file 210_2025_3834_MOESM2_ESM.pptx]

## Slide 1
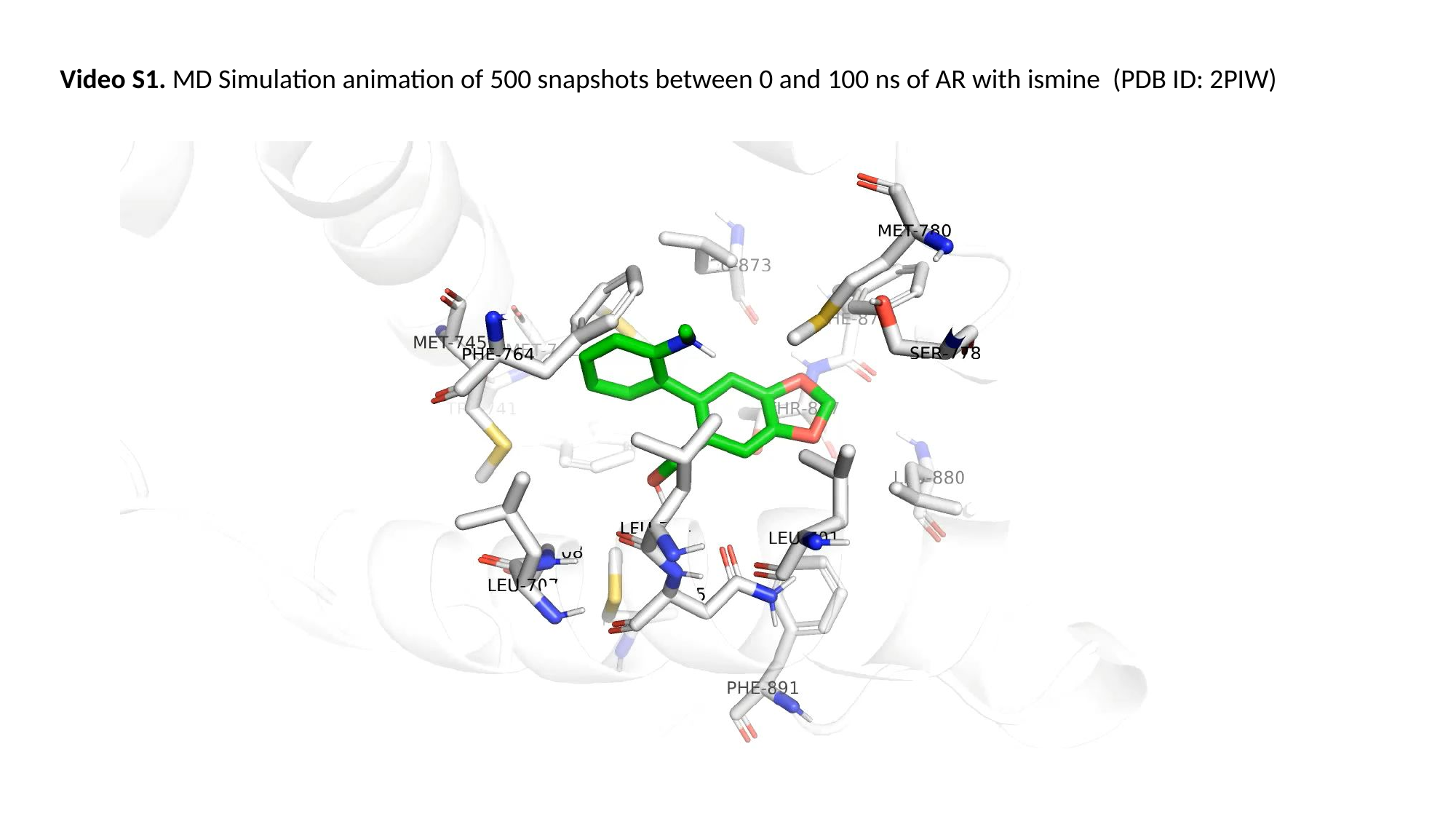

# Video S1. MD Simulation animation of 500 snapshots between 0 and 100 ns of AR with ismine (PDB ID: 2PIW)

## Slide 2
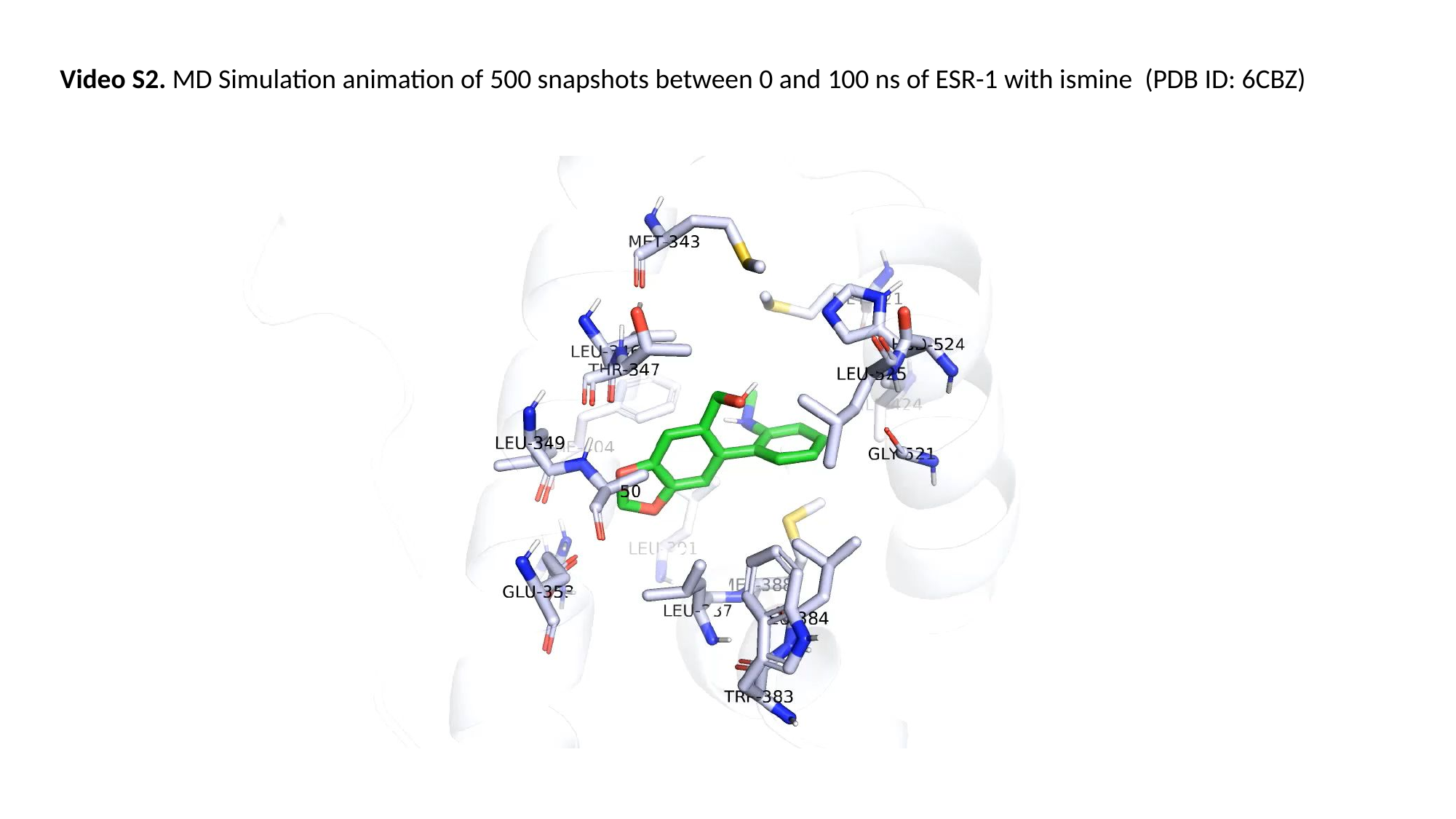

# Video S2. MD Simulation animation of 500 snapshots between 0 and 100 ns of ESR-1 with ismine (PDB ID: 6CBZ)

## Slide 3
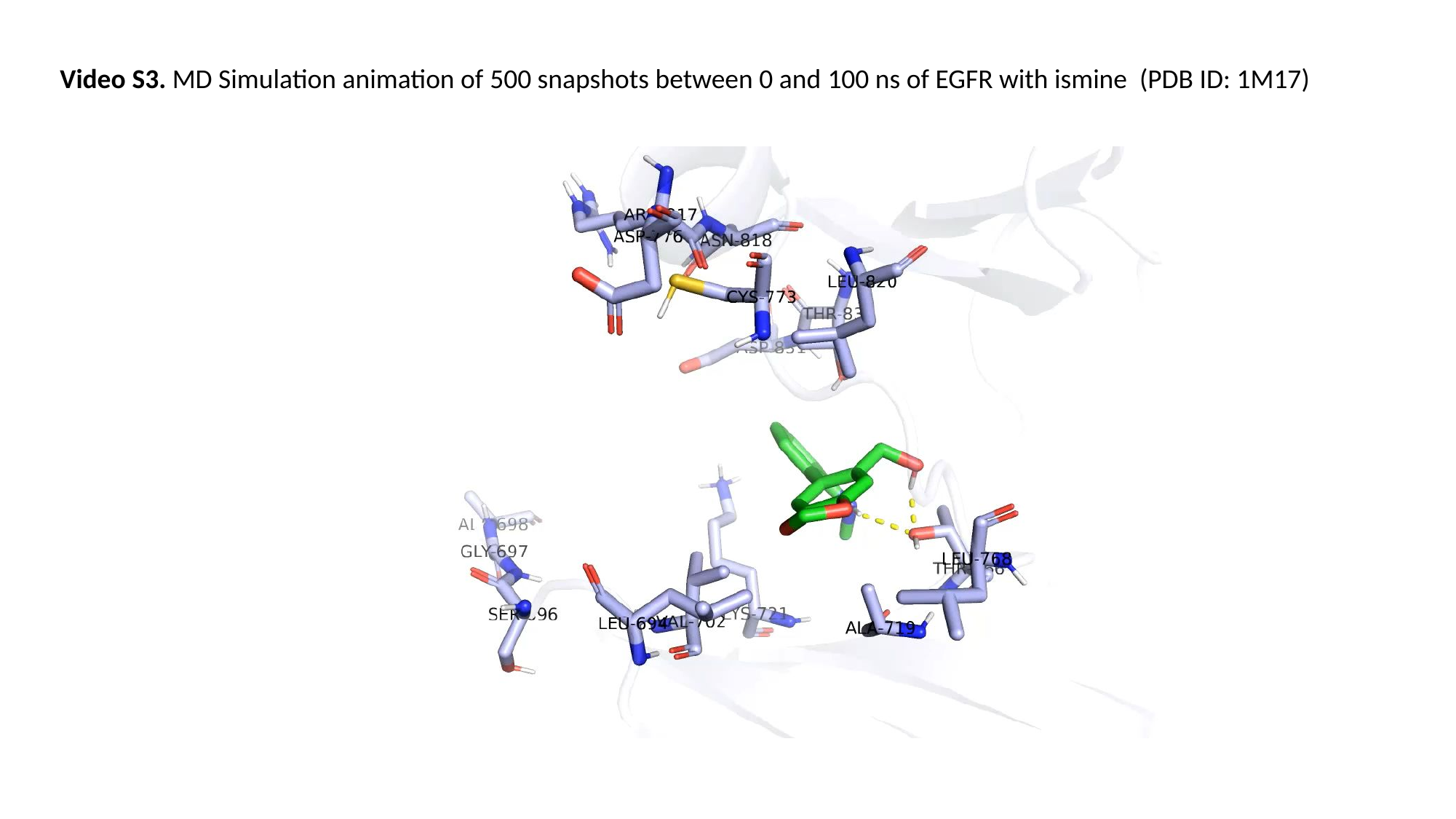

# Video S3. MD Simulation animation of 500 snapshots between 0 and 100 ns of EGFR with ismine (PDB ID: 1M17)
